# Wide‐Temperature Electrolyte Design via Cation‐Anion Solvation Engineering for 4.6 V Lithium‐Ion Batteries

**DOI:** 10.1002/advs.202503151

**Published:** 2025-05-21

**Authors:** Hao Zhang, Yan Zhao, Xiangrong Li, Haoliang Wang, Lu Wang, Yongli Song, Fen Qiao, Junfeng Wang, Jijian Xu

**Affiliations:** ^1^ Department of Chemistry City University of Hong Kong Hong Kong 999077 China; ^2^ School of Energy and Power Engineering Jiangsu University Zhenjiang 212013 China; ^3^ Peking University Shenzhen Graduate School Shenzhen 518055 China; ^4^ School of Energy and Power Engineering Chongqing University Chongqing 400030 China; ^5^ Shenzhen Research Institute City University of Hong Kong Shenzhen 518057 China

**Keywords:** carbonate solvents, CEI, electrolyte design, high‐voltage, lithium‐ion batteries, wide‐temperature

## Abstract

Conventional lithium‐ion batteries (LIBs) employing ethylene carbonate (EC)‐based electrolytes and thermally unstable LiPF_6_ face dual challenges: sluggish Li‐ion transport at low temperatures (≤−20 °C) and severe decomposition at elevated temperatures (≥45 °C). Herein, a synergistic cation‐anion solvation engineering strategy is presented for wide‐temperature electrolytes, combining EC‐free carbonate solvents with a thermally stable ternary lithium salt system. By fine‐tuning solvent‐salt interactions, the designed electrolyte exhibits facilitated desolvation kinetics and superior ionic conductivity under subzero temperatures (0.19 mS cm^−1^ at −60 °C), while also maintaining excellent high‐temperature stability. The anion‐participated solvation structure induces an inorganic‐rich cathode‐electrolyte interphase (CEI), effectively stabilizing the interfacial phase of LiCoO_2_ (LCO) under high voltages. Consequently, the LCO cathode with this electrolyte demonstrates robust performance under wide‐temperature operations. At 4.6 V (versus Li/Li^+^), it retains 88.9% of its capacity after 400 cycles at 25 °C and 77.3% after 200 cycles at 45 °C. Remarkably, a reversible capacity of 110.1 and a discharge capacity of 92.6 mAh g^−1^ are delivered at −35 and −60 °C, respectively, highlighting its exceptional performance under extreme temperatures. This research pioneers a cation‐anion solvation design for tailored electrolytes, enabling reliable LIB operation across a wide temperature range.

## Graphical abstract

This work proposed a synergistic cation‐anion solvation engineering strategy for wide‐temperature and high‐voltage electrolytes. The superior ionic conductivity under subzero temperatures, excellent thermal stability, and induced inorganic‐rich cathode‐electrolyte interphase endow the LiCoO_2_ cathodes reliable performance under harsh conditions with a capacity retention of 88.9% after 400 cycles under 4.6 V operation and reversible capacity of 110.1 mAh g^−1^ at −35 °C.

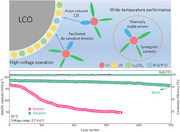

## 1 Introduction

The booming consumer electronics and electric vehicles (EVs) market has triggered surging demand for lithium‐ion batteries (LIBs) with wide temperature performance and high energy density to alleviate range anxiety (e.g., long‐range EVs) and broaden application scenarios (e.g., northeastern China, space exploration, deep‐sea applications).^[^ ^1^, ^2^, ^3^, ^4^ ^]^ However, as the “blood” of LIBs, current commercial electrolytes that rely on ethylene carbonate (EC) and lithium hexafluorophosphate (LiPF_6_) face significant challenges. Though they can function acceptably between 0 and 40 °C at a cutoff voltage below 4.3 V (versus Li/Li^+^), conventional LIBs encounter significant performance limitations under harsher conditions.^[^ ^1^ ^]^ At low temperatures, EC's high viscosity and desolvation energy hinder ion transport, thus limiting the low‐temperature LIB performance.^[^ ^5^ ^]^ Especially, the capacity retention below 0 °C often drops to less than 25% of its room temperature capacity.^[^ ^2^ ^]^ At elevated temperatures (≥45 °C), LiPF_6_ tends to undergo thermal decomposition.^[^ ^6^ ^]^ This leads to the generation of corrosive byproducts, such as phosphorus pentafluoride (PF_5_) and hydrogen fluoride (HF), subsequently triggering solvent decomposition and structural degradation of electrode materials.^[^ ^7^ ^]^ Moreover, the inefficient cathode‐electrolyte interphase (CEI) formed by traditional carbonate‐based electrolytes exacerbates the irreversible phase transition of the cathode material at high voltages. These issues hinder the overall energy density and lifespan of LIBs.^[^ ^8^, ^9^, ^10^, ^11^ ^]^

To address these challenges, tremendous progress has been made in developing novel solvents to improve the wide‐temperature performance and high‐voltage stability of LIBs. For example, the EC‐free electrolyte was proposed by Yao et al., utilizing methyl acetate (MA) and fluorinated ether (FE) as the solvents and realizing 58.3% discharge capacity retention under −60 °C in LIBs operating at 4.2 V (versus Li/Li^+^).^[^ ^12^ ^]^ Then, Xia and co‐workers introduced moderately‐fluorinated ethyl difluoroacetate (EDFA) as the main solvent of electrolyte and achieved a capacity retention of 58.3% at −30 °C.^[^ ^13^ ^]^ More recently, Xu and the co‐workers proposed advanced electrolytes through the design of soft solvents, enabling stable operation across a wide temperature range (±60 °C) under a high voltage operation (4.5 V, versus Li/Li^+^).^[^ ^3^ ^]^ Additionally, nitriles (e.g., isobutyronitrile, fluoroacetonitrile) are considered to be the potential solvents for wide temperature, high voltage LIBs.^[^ ^14^, ^15^ ^]^ Though the application of new solvents has greatly improved the high voltage and wide temperature performance of LIBs, designing advanced electrolytes based on commercialized carbonate solvents is still desirable due to their advantages in manufacturing production.^[^ ^16^ ^]^ Current advanced electrolyte design based on carbonate solvents usually focuses on the physical or chemical properties of a particular solvent, such as low freezing point, low viscosity, or film‐forming ability.^[^ ^17^, ^18^ ^]^ Further research is needed to systematically discuss the properties of carbonate solvents and establish the design criteria of advanced electrolytes on this basis.

**Figure 1:**
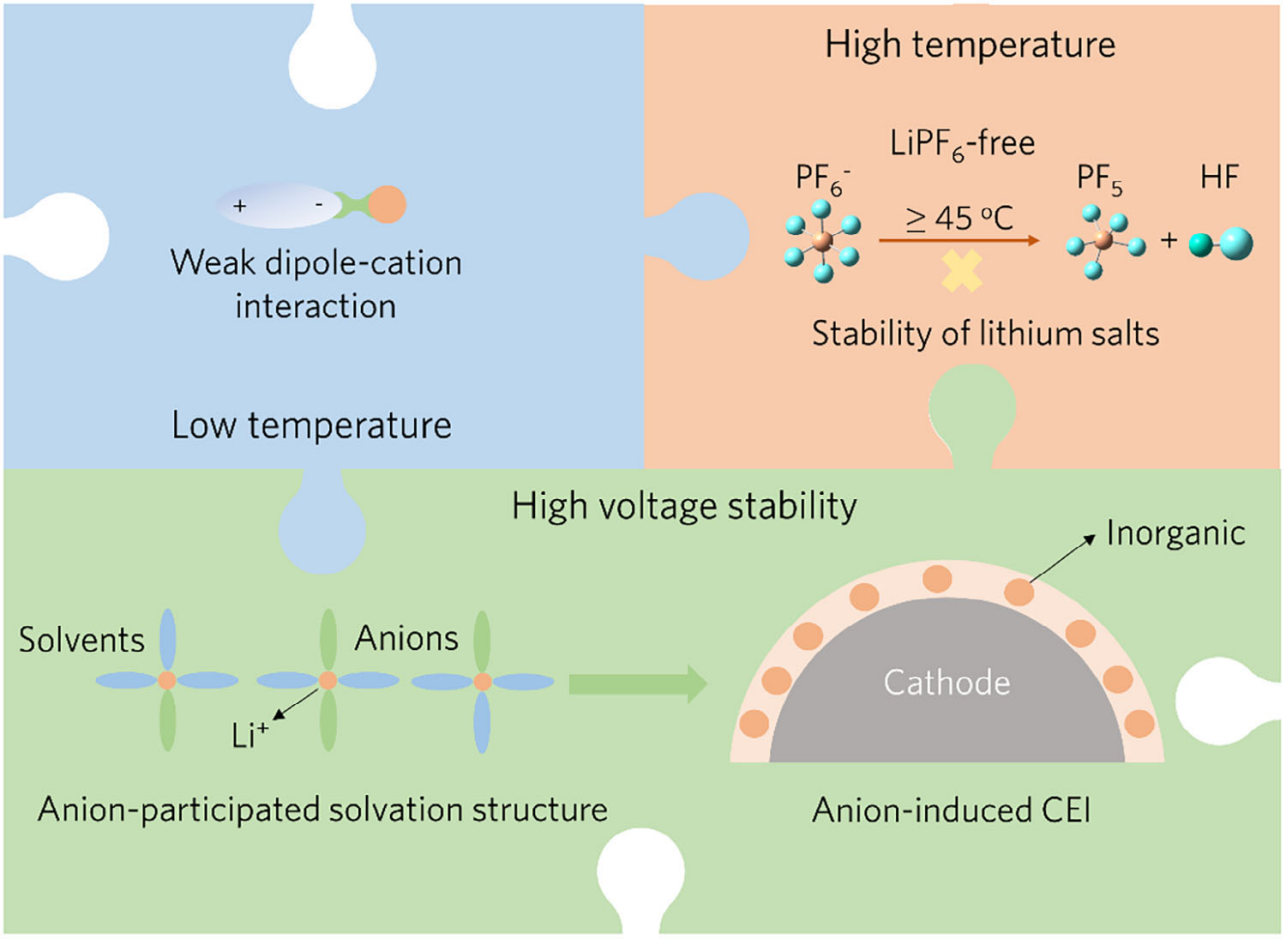
Schematic of the synergistic cation‐anion solvation engineering strategy for the tailored electrolytes, enabling superior performance across a wide temperature range and at high voltages, by controlling solvent‐salt interactions and promoting a protective, anion‐induced CEI.

Among various electrolyte modification methods, tuning the interaction among electrolyte components is crucial for achieving optimal wide‐temperature performance.^[^ ^19^, ^20^ ^]^ Researchers have extensively elucidated the interactions within electrolyte components and their impact on electrochemical performance.^[^ ^21^, ^22^, ^23^, ^24^, ^25^ ^]^ To optimize low‐temperature performance, it is crucial to establish a weak cation‐solvent interaction to minimize the energy barrier for Li^+^ desolvation, while also considering the intrinsic low freezing point of the solvent.^[^ ^26^ ^]^ Additionally, preventing the generation of corrosive HF‐related byproducts and constructing a robust CEI are crucial for enhancing LIB performance under high‐voltage and high‐temperature conditions.^[^ ^27^, ^28^ ^]^ Given these challenges, a carbonate‐based electrolyte tailored for wide‐temperature and high‐voltage applications should encompass the following characteristics (**Figure** **1**): 1) weak cation‐dipole interactions between solvents and Li^+^ to facilitate the Li^+^ desolvation process; 2) excellent thermal stability of the lithium salts to minimize the generation of corrosive byproducts; 3) anion‐participated solvation structure to create a robust, inorganic‐rich CEI to stabilize the cathode interfacial phase during high‐voltage operations.

Hence, in this work, a cation‐anion solvation engineering strategy is proposed to design carbonate‐based electrolytes that simultaneously address low‐temperature ionic conductivity and high‐temperature/high‐voltage stability. The electrolyte integrates a tailored solvent blend—propylene carbonate (PC), ethyl methyl carbonate (EMC), dimethyl carbonate (DMC), and fluoroethylene carbonate (FEC)—with a thermally stable ternary lithium salt system comprising lithium tetrafluoroborate (LiBF_4_), lithium difluorooxalate borate (LiDFOB) and lithium difluorodioxalate phosphate (LiDFBP). This synergistic solvent‐salt optimization is designed to minimize Li^+^‐solvent binding energy and foster anion‐participated Li^+^ solvation structures. The resulting electrolyte exhibits ultrahigh ionic conductivity (0.19 mS cm^−1^ at −60 °C) and fluidity (freezing point <−90 °C) at subzero temperatures, along with significantly reduced Li‐solvent binding energies for fast Li^+^ desolvation. Complementing this, the stronger cation‐anion interactions endow the anion‐participated Li^+^ solvation structure, contributing to an anion‐induced CEI, as evidenced by in situ characterization, enhancing the interfacial stability of the cathode during high‐voltage operations. Ultimately, the designed electrolyte enables the LCO cathode to achieve exceptional electrochemical performance. At 4.6 V (versus Li/Li^+^), the LCO cathode exhibits capacity retentions of 88.9% and 77.3% after 400 and 200 cycles at 25 and 45 °C, respectively. More significantly, a reversible capacity of 110.1 and a discharge capacity of 92.6 mAh g^−1^ are delivered under −35 °C at 0.33 C and under −60 °C at 0.05 C, respectively. These results validate the effectiveness of cation‐anion solvation engineering in decoupling wide‐temperature performance from traditional EC/LiPF_6_ limitations, paving the way for advanced energy storage technologies.

## 2 Results and Discussion

### 2.1 Synergistic Selection of Solvents and Lithium Salts

**Figure 2:**
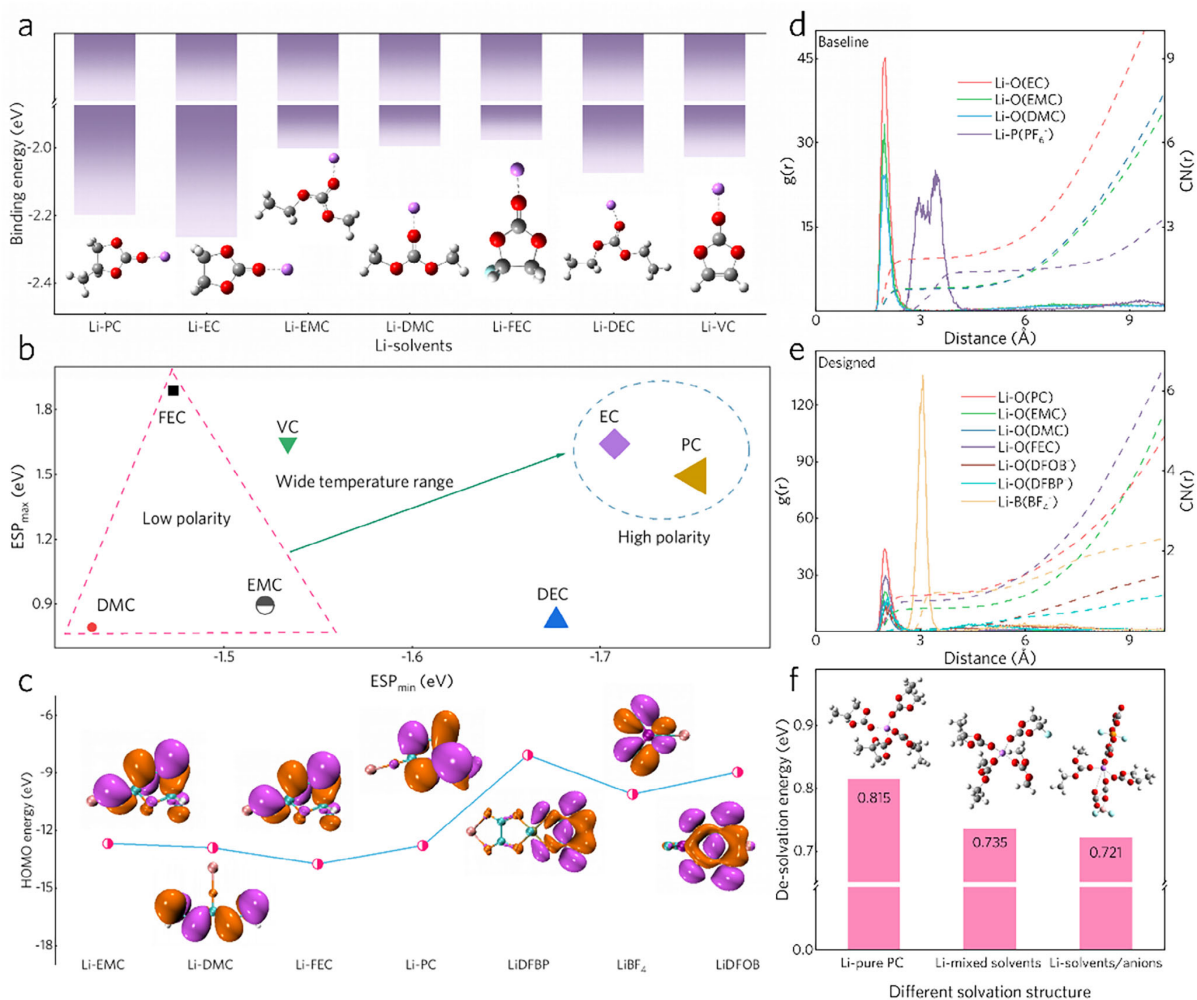
Selection criteria of carbonated solvents and properties of the electrolytes. a) Binding energies of different carbonate solvents with Li^+^. b) Minimum and maximum electrostatic potential (ESP_min_ and ESP_max_) energies of the solvents. c) HOMO energies of the components in the designed electrolyte system. d,e) Radial distribution functions (RDF) and corresponding coordination numbers for (d) the baseline electrolyte and (e) the designed electrolyte. f) Desolvation energies of Li^+^ in different solvation structures.

To optimize the choice of carbonate solvents for wide‐temperature and high‐voltage electrolytes, density functional theory (DFT) calculations were implemented. A weaker dipole‐cation interaction implies the preferred anion‐participated Li^+^ solvation structure and fast Li^+^ desolvation kinetics, which can be inferred by the lower Li‐solvent binding energy.^[^ ^26^ ^]^ Among the commonly used carbonated solvents, EMC, DMC and FEC show relatively lower binding energies with Li^+^ (**Figure** **2**a), theoretically enabling faster Li^+^ desolvation kinetics and thereby enhancing low‐temperature performance. To verify the reliability of this result, three different basis sets were used to conduct the calculation, which show the same distribution trend in Li^+^‐solvent binding energy, indicating the consistency of calculation results (Figure S1, Supporting Information). Moreover, the intrinsic properties of solvents, such as their polarities, were evaluated based on electrostatic potential (ESP) energies. Lower ESP_max_ and ESP_min_ values denote weaker dipole‐cation interactions.^[^ ^29^ ^]^ Considering this, EMC, DMC, and FEC (represented within the triangle in Figure 2b) were identified as favorable solvents for Li^+^ desolvation, consistent with the results of the Li‐solvent binding energies. In addition, the lower Highest Occupied Molecular Orbital (HOMO) energies of these three solvents suggest their enhanced resistance to oxidation at high potentials (Figure S2, Supporting Information). Thus, EMC, DMC, and FEC could act as the primary solvents for an electrolyte designed to operate at both low temperatures and high voltages. However, to ensure the dissolution of lithium salts and maintain the ionic conductivity of the electrolyte, the inclusion of high‐polarity solvents (shown within the circle in Figure 2b) as co‐solvents is essential. Constrained by the high freezing point of EC (Figure S3, Supporting Information), PC was selected as the preferred co‐solvent to improve the wide‐temperature stability of the electrolyte. The physical properties of different carbonate solvents are shown in Table S1 (Supporting Information).^[^ ^30^, ^31^ ^]^ Both EC and PC possess a high dielectric constant, implying their excellent ability to dissolve lithium salts, consistent with their calculated high polarity. Apart from that, low viscosity and freezing point are also essential for enabling the wide temperature performance of LIBs. This endows PC to replace EC as a promising carbonate solvent to ensure the dissociation of lithium salts, while EMC and DMC serve as co‐solvents to reduce the viscosity of the electrolyte. Considering its acknowledged protective effect, FEC is also essential to act as the co‐solvent (Table S2, Supporting Information). After a comprehensive evaluation of various indicators, the solvents PC, EMC, DMC, and FEC were chosen for the designed electrolyte, combining the advantages of wide‐temperature performance, high‐voltage stability, and enhanced ionic conductivity.

Given the stability of lithium salts at elevated temperatures, LiBF_4_, LiDFOB, and LiDFBP were chosen as the main lithium salts for the designed electrolyte to promote the anion‐induced CEI formation and thermal stability of the electrolyte. To substantiate this selection, DFT calculations and molecular dynamics (MD) simulations were executed. In diluent electrolytes, Li^+^ is typically associated with solvent molecules. The CEI in baseline electrolyte predominantly originates from the decomposition of solvents. However, when the DFBP^−^ and DFOB^−^ anions bind to Li^+^, their elevated HOMO energies suggest that the designed electrolyte has a high potential to form anion‐induced CEI via an anion‐involved Li^+^ solvation structure (Figure 2c). To further investigate this, MD simulations were performed for both baseline and designed electrolytes. For the baseline electrolyte, solvents predominantly constitute the first solvation sheath of Li^+^ (Figure 2d). In contrast, the designed electrolyte showcases a Li^+^ solvation structure that involves DFOB^−^ and DFBP^−^, facilitating the formation of an anion‐induced CEI (Figure 2e). This change in the solvation structure is attributed, on the one hand, to the relatively low Li^+^‐solvent binding energies of the tailored solvents. On the other hand, the stronger cation‐anion interactions provided by the selected lithium salts with high thermal stability further promote the formation of this unique anion‐involved solvation structure (Figure S4, Supporting Information). The ^7^Li nuclear magnetic resonance (NMR) spectra confirm the modified Li^+^ solvation structure of the designed electrolyte (Figure S5, Supporting Information). Compared with the baseline electrolyte, the ^7^Li NMR spectra of the designed electrolyte slightly shift to a lower frequency, indicating an enhanced electron‐cloud density around Li^+^, mainly caused by the binding with anions.^[^ ^32^ ^]^ The formation of anion‐induced CEI is evidenced by the preferential oxidation peak observed in the linear sweep voltammetry (LSV) profiles of the designed electrolyte (Figure S6, Supporting Information). Moreover, using a cutoff current density of 4 uA cm^−2^, the decomposition voltage of the designed electrolyte is 4.43 V (versus Li/Li^+^), higher than that of the basic electrolyte (4.22 V, versus Li/Li^+^), demonstrating the higher antioxidant ability of the designed carbonate‐based electrolyte. Moreover, to reveal the effect of dipole‐cation interactions on Li^+^ desolvation kinetics, three different solvation structures of Li^+^ were evaluated (Figure 2f). Initially, the Li^+^ desolvation energy is highest when Li^+^ is bound to the pure solvent (PC) with high polarity, and this energy decreases as weakly polarized solvents (FEC/EMC) participate in the solvation structure. The electrostatic attraction between anions and Li^+^ weakens the cation‐dipole interaction, which in turn lowers the energy barrier for Li^+^ desolvation and enhances Li^+^ interfacial transfer kinetics. These results indicate the importance of designing the weak dipole‐cation interactions and anion‐involved Li^+^ solvation structure to enhance Li^+^ desolvation kinetics.

### 2.2 Physicochemical Properties of the Electrolytes

**Figure 3:**
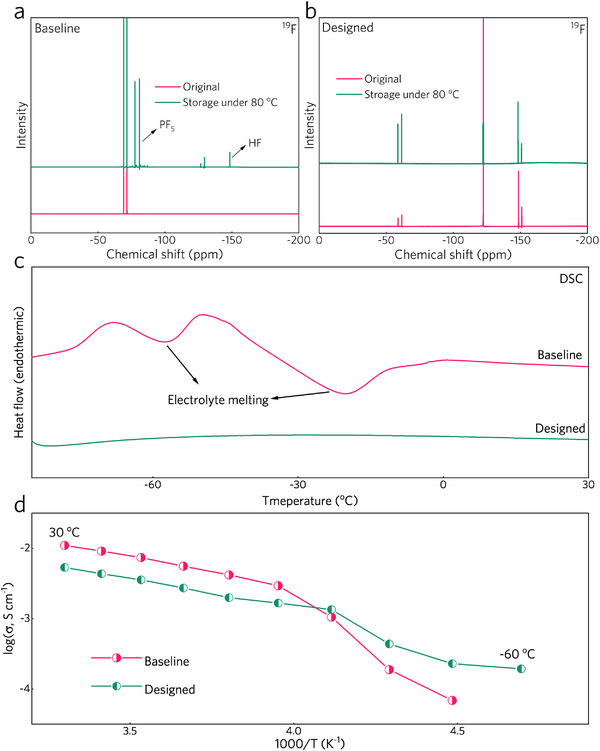
Electrolyte properties. a,b) ^19^F NMR spectra of (a) baseline and (b) designed electrolytes before and after 144 h of storage. c) DSC profiles from 30 to −85 °C. d) Temperature‐dependent ionic conductivities of baseline and designed electrolytes.

To investigate the potential application of the designed electrolyte in wide‐temperature LIBs, the electrolyte's thermal stability and temperature‐dependent ionic conductivity were evaluated. First, the designed electrolyte demonstrated negligible thermal decomposition under high temperatures due to the lithium salts with high thermal stability. In contrast, the baseline electrolyte with LiPF_6_ showed significant degradation: after 144 h of storage at 80 °C, the electrolyte turned brown, and the characteristic ^19^F NMR peaks of PF_5_ and HF were detected (**Figure** **3**a).^[^ ^33^ ^]^ This can be attributed to the severe decomposition of LiPF_6_ under high‐temperature conditions. Meanwhile, new ^1^H NMR characteristic peaks, aside from the solvent peaks, appeared in the baseline electrolyte after long‐term high‐temperature storage, suggesting products from the reaction between carbonate solvents and PF_5_ (Figure S7, Supporting Information). These results demonstrate the poor thermal stability of LiPF_6_‐based electrolytes. Conversely, no discernible decomposition products were observed in the designed electrolyte, showing its superior thermal stability and potential for high‐temperature applications (Figure 3b).

Differential scanning calorimetry (DSC) profiles in Figure 3c demonstrated that, during the heating process from −85 to 30 °C, the baseline electrolyte showed endothermic peaks located at −57.1 and −20.1 °C, indicating the solid‐liquid phase transformation. Surprisingly, no endothermic peak was observed for the designed electrolyte, indicating it remained in liquid form across the temperature range from −80 to 30 °C. This behavior is attributed to the judicious selection of solvent blends. The optical photos of the electrolytes at different temperatures further support this: while the baseline electrolyte solidified at −60 °C, the designed electrolyte remained liquid at −90 °C (Figure S8, Supporting Information). Owing to the extremely low freezing point and the moderate lithium salt dissociation ability of the rationally selected solvents, the designed electrolyte exhibits exceptional ionic conductivity at reduced temperatures (Figure 3d). Its high ionic conductivity (0.19 mS cm^−1^ at −60 °C) indicates its potential for applications in low‐temperature environments.

### 2.3 Electrochemical Performance of the Electrolytes

**Figure 4:**
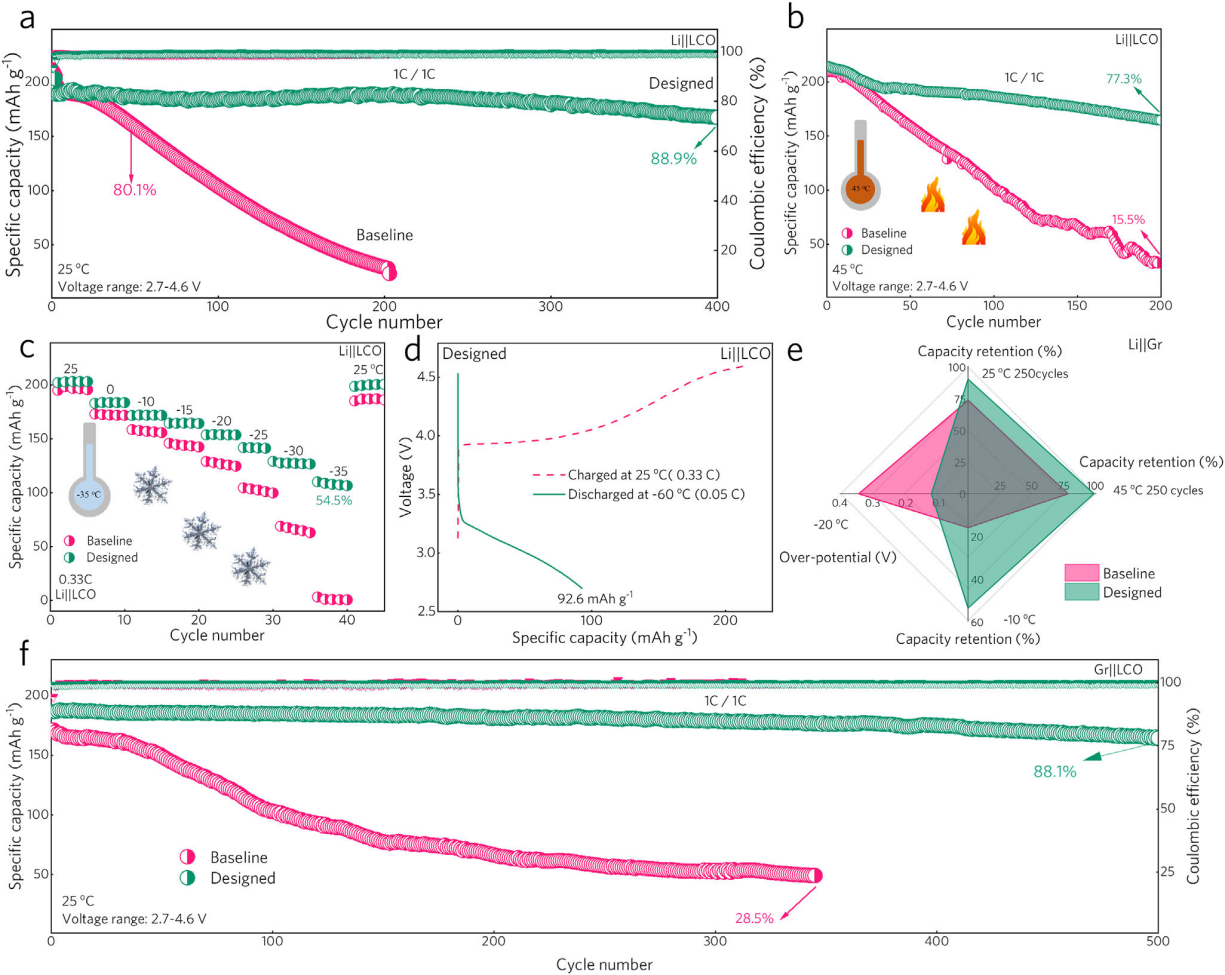
Electrochemical performance of baseline and designed electrolytes. a) Long‐term cycling performance of Li||LCO half‐cells at 25 °C (2.7–4.6 V versus Li/Li^+^). b) Cycling performance of Li||LCO half‐cells at 45 °C. c) Capacity retention of Li||LCO half‐cells under different temperatures at 0.33 C. d) Discharge capacity exhibition of Li||LCO half‐cell using designed electrolyte under −60 °C at 0.05 C after charged under 25 °C. e) Electrochemical performance of Li||graphite half‐cells. f) Long‐term cycling performance of graphite||LCO full‐cells at 1 C and 25 °C.

The combination of high thermal stability, excellent low‐temperature ionic conductivity, and the capability to form an anion‐induced CEI endows the designed electrolyte with high application potential for wide‐temperature, high‐voltage LIBs. To verify this, the electrochemical performance of both LCO and graphite electrodes was tested. With an upper cut‐off voltage at 4.6 V (versus Li/Li^+^), the LCO cathodes cycled in both electrolytes deliver a high reversible capacity of ≈190 mAh g^−1^ with the current density at 1 C after activation. Interestingly, the Li||LCO half‐cells using the designed electrolyte achieve excellent long‐term cycling stability, retaining 88.9% capacity over 400 cycles at 25 °C (**Figure** **4**a). And the excellent long‐term cycling exhibits high reproducibility, as shown in Figure S9 (Supporting Information). Li||LCO half cells using different batches of designed electrolyte all exhibit stable long‐term cycling performance with a standard deviation of 4.1% after 200 cycles. In contrast, the LCO electrode in the baseline electrolyte undergoes dramatic capacity decay and severe charging/discharging plateau degradation (Figure S10, Supporting Information), with only a reversible capacity of 23.4 mAh g^−1^ after 200 cycles. Moreover, Li||LCO half cells using the designed electrolyte achieve an average Coulombic efficiency (CE) up to 99.4% (Figure S11, Supporting Information), higher than that of the baseline electrolyte (99.03%). The slightly lower CE observed during the initial activation cycles with the designed electrolyte can be attributed to the anion‐induced CEI formation process, which quickly forms a dense CEI on the LCO surface. These results indicate that the CEI formed by the baseline electrolyte cannot protect LCO from the irreversible phase transformation under high voltages.

Under elevated temperature conditions (45 °C), the LCO in the baseline electrolyte shows poor cycling performance, retaining only 15.5% capacity after 200 cycles and exhibiting low, fluctuating average CE. This poor performance is primarily due to the unstable CEI formed by the baseline electrolyte. In contrast, owing to the outstanding thermal stability of the designed electrolyte and the formation of anion‐induced CEI, LCO cathode with designed electrolyte demonstrates 77.3% capacity retention after 200 cycles at 45 °C (Figure 4b), exhibiting stable discharge voltage and high average CE of up to 99.35% (Figure S12, Supporting Information). More importantly, the high ionic conductivity of the designed electrolyte below 0 °C enables the LCO cathode to deliver high capacity under low temperatures (Figure 4c). Notably, baseline electrolyte delivers reversible capacities of 198.4, 171.8, 156.4, 142.5, 124.6, 101.0, 62.9, and 0.59 mAh g^−1^ at 25, 0, −10, −15, −20, −25, −30, and −35 °C, respectively, indicating that the baseline electrolyte is nearly inactive at −35 °C. On the contrary, the designed electrolyte endows the LCO cathode to deliver reversible capacities of 202.1, 183.6, 171.9, 164.5, 154.1, 141.3, 129.1, and 110.1 mAh g^−1^ with the same temperature gradient and a current density at 0.33 C. This corresponds to a significantly high capacity retention of 54.5% at −35 °C (compared with the capacity at 25 °C), clearly demonstrating the wide‐temperature advantage of the designed electrolyte. Even at −60 °C, a high discharge specific capacity of 92.6 mAh g^−1^ is delivered by the LCO cathode in the designed electrolyte at a current density of 0.05 C (Figure 4d). The over‐potential of the Li||LCO half‐cells using different electrolytes accounts for the significant performance difference, as shown in Figure S13 (Supporting Information). The over‐potential of the Li||LCO half cells using the baseline electrolyte exceeds 0.7 V (versus Li/Li^+^) at −35 °C, about three times that observed with the designed electrolyte. Such a large overpotential is considered the result of the extremely low ionic conductivity at low temperatures and the sluggish Li^+^ desolvation process of the baseline electrolyte.

Furthermore, the graphite anode with the designed electrolyte also exhibits excellent electrochemical performance (Figure 4e). At both 25 and 45 °C, the graphite anode exhibits improved cycling stability in the designed electrolyte, with negligible capacity decay (Figure S14, Supporting Information). Especially at −10 °C, due to the significantly reduced overpotential, graphite cycling in the designed electrolyte exhibits 212.5 mAh g^−1^ with a current density at 0.2 C, ≈3 times the capacity exhibited by that cycling in the baseline electrolyte (Figure S15, Supporting Information). These results demonstrate the potential of the designed electrolyte for use in full‐cells. Consequently, the assembled full‐cells using the designed electrolyte deliver a higher specific capacity of 186.6 mAh g^−1^ and exhibit superior cycling stability. Specifically, the capacity retention reaches 88.1% over 500 cycles at 4.6 V (versus Li/Li^+^). To further demonstrate the reproducibility, full‐cells using different batches of designed electrolytes were assembled, which all exhibit high capacity retention with a standard deviation of only 3.8% after 250 cycles (Figure S16, Supporting Information). Furthermore, the corresponding CE rises to 99.0% after two activation cycles (Figure S17, Supporting Information), suggesting minimal electrolyte decomposition. This can be attributed to the excellent CEI‐forming ability of the designed electrolyte. As mentioned earlier, the LCO cathode will exhibit a high energy density under the elevated charging cutoff voltage. With the cutoff voltage at 4.6 V (versus Li/Li^+^), theoretically, the gravimetric and volumetric energy density of the LCO cathode can reach 885.9 Wh kg^−1^ and 3721 Wh L^−1^, respectively.^[^ ^34^ ^]^ Matched with the graphite anode, the projected energy density of the assembled graphite||LCO full‐cells was estimated to be 303 Wh Kg^−1^,^[^ ^35^ ^]^ with the corresponding parameters summarized in Table S3 (Supporting Information). These results highlight the high application potential of the designed electrolyte in real‐world LIBs.

### 2.4 Properties of Different CEI

**Figure 5:**
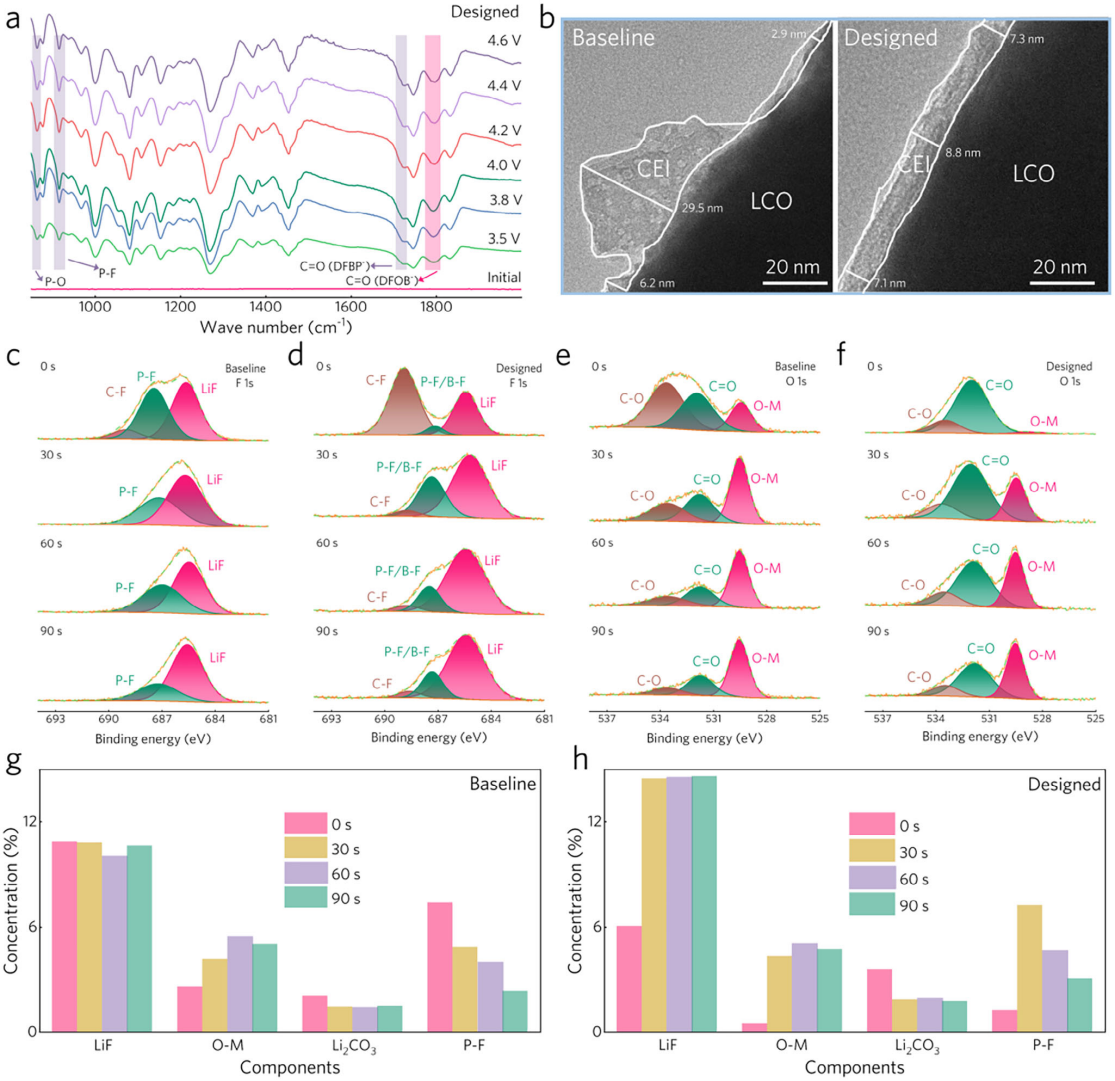
Characterization of CEI. a) In situ FTIR spectra of the designed electrolyte during the first cycle. b) TEM images of LCO particles after 50 cycles with different electrolytes. c,d) F *1s* spectra of LCO surface after 50 cycles in (c) baseline electrolyte and (d) designed electrolyte. e,f) O *1s* spectra of LCO surface after 50 cycles in (e) baseline electrolyte and (f) designed electrolyte. g,h) Concentrations of selected components on LCO surface after 50 cycles in (g) baseline electrolyte and (h) designed electrolyte with different etching times.

Given the excellent cycling stability of the LCO cathode under high voltages, which is expected to be contributed by the anion‐induced CEI, in situ Fourier transform infrared spectrometer (FTIR) was conducted to confirm this.^[^ ^36^ ^]^ Figure S18 (Supporting Information) shows the ex situ FTIR spectra of pure EMC, EMC solutions with different lithium salts, and the designed electrolyte. Specifically, the characteristic peak of LiBF_4_ is observed at 1063.1 cm^−1^, corresponding to the B‐F stretching vibration mode, while LiDFOB's characteristic peak is found at 1794.2 cm^−1^, assigned to the C═O stretching vibration.^[^ ^37^, ^38^ ^]^ For LiDFBP, more characteristic peaks appear, which are located at ≈862.1, 915.2, and 1715.3 cm^−1^, representing P‐F, P‐O, and C═O stretching vibrations, respectively.^[^ ^39^, ^40^, ^41^ ^]^ When charged to 3.5 V (versus Li/Li^+^), five characteristic peaks of oxidation appeared, corresponding to the decomposition of P‐O bonds (863.9 cm^−1^), P‐F bonds (915.5 cm^−1^), and the C═O bond of DFOB^−^ (1793.5 cm^−1^) as well as DFBP^−^ (1716.3 cm^−1^), indicating the oxidation of DFOB^−^ and DFBP^−^ to form CEI (**Figure** **5**a). The slight difference in the characteristic peak of certain lithium salts in the EMC solution and the designed electrolyte is attributed to the interaction between components in the electrolyte. In particular, the characteristic peak of BF_4_^−^ oxidation did not appear even when charged to 4.6 V (versus Li/Li^+^), indicating that it is difficult for BF_4_^−^ to participate in the formation of CEI in the designed electrolyte.

To analyze the morphology and composition of anion‐induced CEI, scanning electron microscope (SEM), high‐resolution transmission electron microscope (HR‐TEM), and X‐ray photoelectron spectroscopy (XPS) were carried out. Due to the enhanced CEI formation ability, the designed electrolyte manifests efficient construction of a protective interphase on the LCO surface after the first two activation cycles, as shown in the SEM images (Figure S19, Supporting Information). More specifically, the TEM patterns demonstrate the dense and uniform morphology of the CEI induced by the designed electrolyte with a thickness of ≈7–8 nm (Figure 5b). In sharp contrast, an uneven CEI is observed on the surface of LCO cycled in baseline electrolyte, with the thickness ranging from several nanometers to ≈30 nm.

Then, XPS data reveal the composition of the CEI formed by different electrolytes. LiF, known for its high chemical inertness, is considered essential in suppressing the interfacial degradation of layered cathodes during high‐voltage operations.^[^ ^42^, ^43^, ^44^, ^45^, ^46^, ^47^, ^48^ ^]^ Due to the oxidative decomposition of anions, the CEI formed by the designed electrolyte is richer in F elements (Figure S20, Supporting Information). In addition, the proportion of LiF (located at 685.5 eV) in the CEI formed by the baseline electrolyte decreases with the increase of etching time (Figure 5c; Figure S21, Supporting Information), while a larger proportion of LiF generated by the designed electrolyte is distributed in the inner layer of CEI (Figure 5d; Figure S22, Supporting Information), which benefits from the preferential oxidation of the anions. More LiF in the inner CEI, which is closer to the LCO surface, makes it more effective in suppressing the irreversible LCO interfacial phase transition.^[^ ^49^, ^50^ ^]^ The higher proportion of C‐F bonds on the surface of LCO cathode cycled in designed electrolyte is mainly attributed to the partial exposure of the polyvinylidene difluoride (PVDF) binder caused by the thinner CEI, which can also be supported by the gradient of high atomic percentages for Co element on the LCO surface (Figure S20, Supporting Information). Moreover, the P‐O and B‐O species generated by the designed electrolyte further enhance the CEI stability, inhibiting the continuous electrolyte decomposition, especially under high temperatures (Figure S23, Supporting Information). The O1s XPS spectra also demonstrate the dense nature of CEI formed by the designed electrolyte. The O‐M species (≈529.5 eV) is considered as the Co‐O bonds, mainly contributed by the LCO particle.^[^ ^51^, ^52^ ^]^ While on the surface without etching, the observed O‐M species may partially arise from dissolved Co ions.^[^ ^53^ ^]^ Therefore, the relatively higher proportion of O‐M species on the LCO surface cycled in baseline electrolyte indicates that the formed CEI cannot effectively inhibit the dissolution of Co ions (Figure 5e; Figure S24, Supporting Information). Notably, the relatively higher content of C═O bonds observed on the LCO surface cycled in the designed electrolyte may stem from the higher proportion of lithium carbonate (Li_2_CO_3_) in the induced CEI (Figure 5f; Figure S25, Supporting Information), which can be verified by the C 1s XPS spectra (Figure S26, Supporting Information). Figure 5g,h show the specific concentration gradient of certain components in different CEI layers. With increasing etching depth, the concentration of inorganic components in CEI induced by the designed electrolyte is higher, including LiF, Li_2_CO_3_, and P‐F species, revealing the ability to form the inorganic‐rich CEI of the designed electrolyte. These findings reinforce the superior interfacial stability offered by the designed electrolyte under high‐voltage operation.

### 2.5 Stability of CEI and Protection on the Interfacial Phase of LCO

To reveal the stability of different CEI, HR‐TEM and XPS were conducted after 200 cycles. As shown in Figure S27 (Supporting Information), after prolonged cycling, the thickness of CEI induced by the designed electrolyte increases to ≈10–20 nm while maintaining a dense and uniform morphology. However, the CEI on the LCO surface formed in the baseline electrolyte exhibits significant structural degradation, with visible breakdowns and partial exposure of the underlying LCO particles. This phenomenon is attributed to the fact that the higher mechanical strength of the anion‐induced CEI with more inorganic components enhances its stability during the long‐term cycling process. Moreover, the CEI induced by the designed electrolyte exhibits more F element and less C element, retaining its composition features (Figure S28, Supporting Information), while there is more Co element on the LCO surface after cycling in the baseline electrolyte, demonstrating the partial CEI cracks as shown in TEM patterns in Figure S27 (Supporting Information). These results demonstrate the high stability, morphologically and chemically, of the protective interphase formed by the designed electrolyte on the LCO surface.

**Figure 6:**
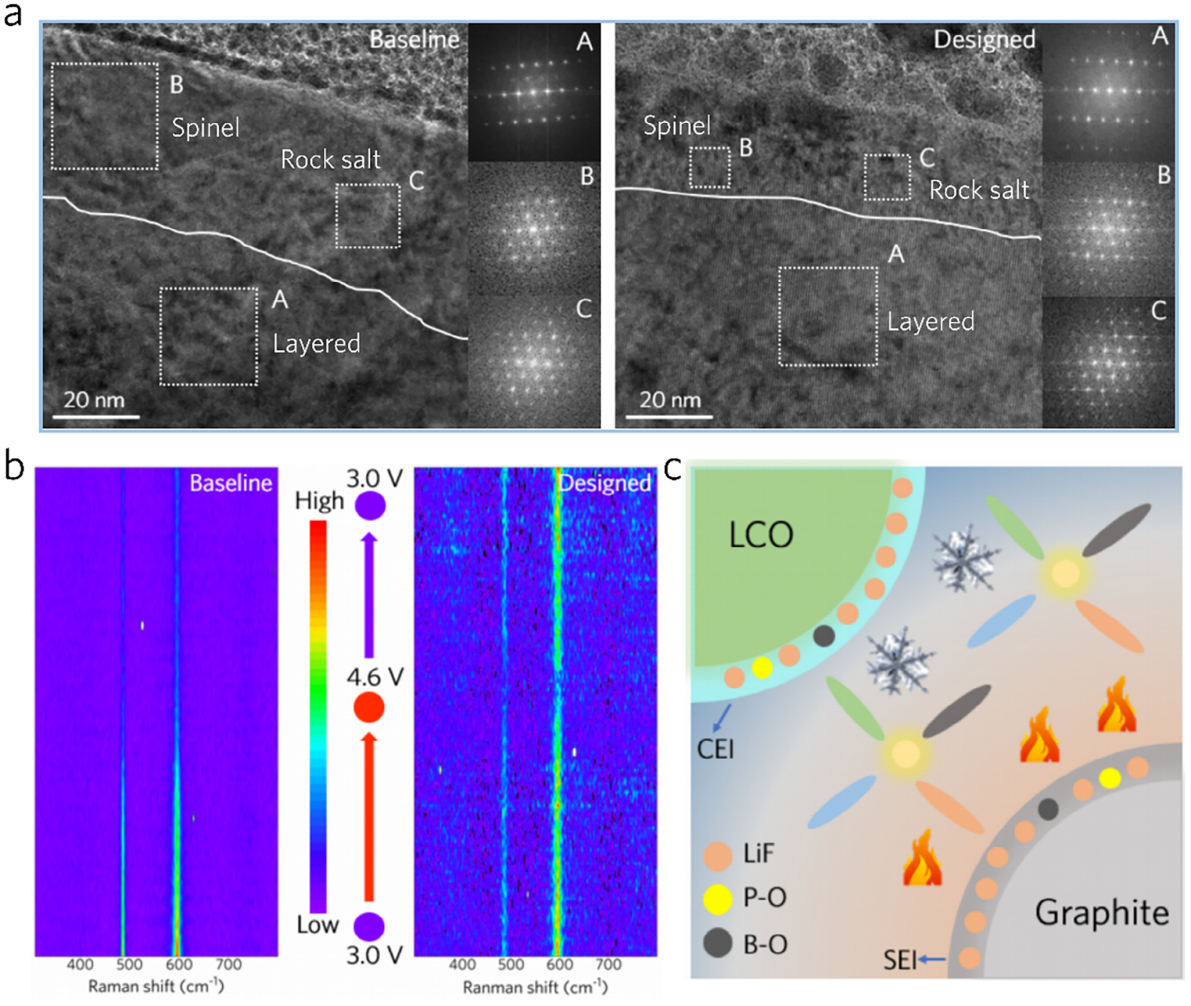
Protection of the LCO interface with the designed electrolyte. a) HR‐TEM images of the LCO surface after 200 cycles with the baseline and designed electrolytes, including corresponding FFT patterns. b) In situ Raman spectra of LCO particles during the first cycle using different electrolytes. c) Schematic of the mechanism by which the designed electrolyte improves the electrochemical performance of the graphite||LCO full‐cells.

The protective role of the designed electrolyte on the LCO interfacial phase under high voltages was investigated using HR‐TEM and in situ Raman. First, the HR‐TEM images and corresponding fast Fourier transform (FFT) patterns of cycled LCO particles confirm the protective role of the designed electrolyte on the LCO interfacial structure (**Figure** **6**a). After 200 cycles in the baseline electrolyte, regions exhibiting spinel and rock salt phases with a thickness of ≈40 nm are observed on the LCO surface. Conversely, the LCO particles cycled in the designed electrolyte predominantly retain the layered structure, confirming that anion‐induced CEI can effectively inhibit the interfacial structure degradation of LCO under high voltage. In situ Raman was used to monitor the structural evolution of the LCO interfacial phase during cycling (Figure 6b). At the onset of charging, two characteristic peaks (485 and 595 cm^−1^, O‐Co‐O bending (E_g_) and Co‐O stretching (A_1g_)) appear for LCO in both electrolytes, indicating the layered phase.^[^ ^54^ ^]^ These peaks disappear when the charging voltage reaches 4.6 V (versus Li/Li^+^),^[^ ^55^ ^]^ indicating the phase transition of LCO from the layered phase to the spinel phase at high voltage. However, when discharged to 3.0 V (versus Li/Li^+^), the characteristic peaks of the layered phase fail to reappear for cycled LCO in the baseline electrolyte, whereas cycled LCO in the designed electrolyte fully reverts to the layered phase. This reconfirms the enhanced effect of the designed electrolyte in promoting the reversible phase transition of LCO at high voltages.

By combining high‐temperature stability and enhanced ionic conductivity under low temperatures, the designed electrolyte enables robust high‐voltage operation across a wide temperature range (Figure 6c). Specifically, the alleviated side‐reaction, including continuous decomposition of electrolyte, irreversible interfacial phase transition of LCO, the thermal decomposition of electrolyte, and inevitable CEI evolution, endows the LCO cathodes with significantly enhanced cycling stability under high cutoff voltage and elevated temperatures. These improvements are contributed by the anion‐induced dense protective interphase with high chemical inertness and uniform morphology, as well as the inherent antioxidant property and thermal stability of the designed electrolyte. Moreover, the low over‐potential imparted by the designed electrolyte and its inherent high ionic conductivity at low temperatures enable the battery to exhibit considerable capacity at extremely low temperatures. This demonstrates the great potential of cation‐anion solvation engineering in designing advanced electrolytes capable of delivering stable and reliable performance under harsh conditions.

## 3 Conclusion

In summary, to develop wide‐temperature and high‐voltage electrolytes based on carbonate solvents, we proposed a synergistic cation‐anion solvation engineering strategy that focuses on tuning the interaction between the electrolyte components and optimizing lithium salts. Concerning the multidimensional indicators, PC, EMC, DMC, and FEC were selected as co‐solvents, featuring a disrupted Li^+^ solvation structure. For lithium salts, LiBF_4_, LiDFOB, and LiDFBP were selected for their high‐temperature stability, ionic conductivity, and the ability to form an anion‐induced CEI. The designed electrolyte, with an extremely low freezing point (<−90 °C), endows fast Li^+^ transport within the bulk electrolyte, while the weak cation‐solvent interaction minimizes the energy barrier for Li^+^ desolvation. Replacing LiPF_6_ with the ternary lithium salts (LiBF_4_, LiDFOB, and LiDFBP) not only enhances the thermal stability but also promotes the formation of anion‐induced CEI rich in LiF. As a result, the designed electrolyte demonstrates high ionic conductivity and stability over a wide temperature range. LCO cathodes using the designed electrolyte exhibit excellent long‐term cycling stability, with capacity retention rates of 88.9% and 77.3% after 400 and 200 cycles at 25 and 45 °C, respectively. Furthermore, a reversible capacity of 110.1 and a discharge capacity of 92.6 mAh g^−1^ are obtained even at −35 and −60 °C, respectively. The high capacity retention of up to 88.1% after 500 cycles at 4.6 V (versus Li/Li^+^), as observed in graphite||LCO full‐cells, affirms the practical applicability of the designed electrolyte. Our research provides insights for the development of high‐practicability, wide‐temperature electrolytes with high stability for high‐voltage operations.

## Conflict of Interest

The authors declare no conflict of interest.

## Author Contributions

H.Z. conducted the experiments, analyzed the data, and wrote the manuscript. X.L. conducted the DSC experiment. H.W. conducted the in situ FTIR experiment. L.W. conducted the in situ Raman experiment. Y.S., F.Q., and J.W. analyzed the experimental data. J.X. and Y.Z. conceived and supervised the project. All the authors contributed to the interpretation of the results.

## Supporting information



Supporting Information

Supporting Information

## Data Availability

The data that support the findings of this study are available from the corresponding author upon reasonable request.
