# Supplementary material for: Wide‐Temperature Electrolyte Design via Cation‐Anion Solvation Engineering for 4.6 V Lithium‐Ion Batteries
Source: Adv Sci (Weinh). 2025 May 21;12(32):e03151. doi: 10.1002/advs.202503151 (PMC12407330; doi:10.1002/advs.202503151)
Supplement: Supplementary file 1 — Supporting Information [file ADVS-12-e03151-s001.docx]

**Supporting information**

**Wide-Temperature Electrolyte Design via Cation-Anion Solvation Engineering for 4.6 V Lithium-Ion Batteries**

Hao Zhang^1^, Yan Zhao^2*^, Xiangrong Li^1^, Haoliang Wang^3^, Lu Wang^3^, Yongli Song^2^, Fen Qiao^2^, Junfeng Wang^2,4^, Jijian Xu^1,5*^

^1^ Department of Chemistry, City University of Hong Kong, Hong Kong, China

E-mail: [jijianxu@cityu.edu.hk](mailto:jijianxu@cityu.edu.hk)

^2^ School of Energy and Power Engineering, Jiangsu University, Zhenjiang 212013, China

E-mail: [zhaoyan@ujs.edu.cn](mailto:zhaoyan@ujs.edu.cn)

^3^ Peking University Shenzhen Graduate School, Shenzhen 518055, China

^4^ School of Energy and Power Engineering, Chongqing University, Chongqing, 400030 China

^5^ Shenzhen Research Institute, City University of Hong Kong, Shenzhen, 518057, China

^*^ Corresponding authors

**Experimental section**

1. Material preparation

LCO electrode was purchased from WeiMing Co., Ltd., China. Graphite material was purchased from Shenzhen BTR Tech Co., Ltd., China. The baseline electrolyte consisting of 1.0 M LiPF_6_ in EC/EMC/DMC=1/1/1, by vol. was purchased from DodoChem. The solvents of PC, EMC, DMC, FEC and the lithium salts of LiBF_4_ and LiDFOB were purchased from DodoChem. The lithium salt of LiDFBP was purchased from the Changsha T-rock Biochem Co., LTD. The designed electrolyte comprised 0.4 M LiBF_4_, 0.4 M LiDFOB and 0.3 M LiDFBP in PC/EMC/DMC/FEC=2/3/3/2, by vol. The cathode for half-cells comprised 90 wt.% LCO, 5 wt.% Super P and 5 wt.% polyvinylidene fluoride (PVDF) while the anode comprised 70 wt.% graphite, 20 wt.% Super P, 4 wt.% sodium carboxymethyl cellulose (CMC) and 6 wt.% styrene butadiene rubber (SBR). The mass loadings of the cathode and anode for half-cells were 6.6 and 2.0 mg cm^-2^, respectively. The mass loading of the cathode and anode for full-cells were 6.6 and 4.0 mg cm^-2^ respectively. The anode for full-cells was composed of 94.5 wt% graphite, 2.5 wt% Super P, 1.5 wt% CMC and 1.5 wt% SBR.

2. Electrochemical measurements

Coin cells were assembled by CR2032 in the argon-filled glovebox with Celgard 2400 (19 mm diameter) as the separator. 80 ul of electrolyte was added to each cell. The assembled coin cells were tested on a NEWARE battery test system. For the long-cycling performance, the coin cells were tested using the constant current charging and discharging profiles and 10 s rest was implemented after every step. Liner sweep voltammetry (LSV) was tested on the Solartron Analytical 1470E electrochemical workstation with a scan rate of 0.5 mV s^-1^. The ionic conductivity of different electrolyte was calculated by the Rs impedance of the three-electrode model under different temperatures. The ionic conductivity of the electrolytes was calculated using the following formula:

$$\sigma=a/Rs$$

where σ represents the ionic conductivity, Rs is the measured impedance and a is the constant which is obtained by the subsequent formula:

$$a=\sigma^{'}*Rs'$$

where σ’ is the ionic conductivity of the baseline electrolyte at 25 ^o^C obtained from the seller and Rs’ is the impedance of the baseline electrolyte at 25 ^o^C. The material of the three electrodes is titanium with an area of 0.785 cm^-2^. EIS was conducted over a frequency range of 1000 kHz to 0.1 Hz with an AC signal amplitude of 10 mV. To ensure accurate temperature control and thermal equilibration, the assembled cells were stored at the target temperature for at least four hours before the EIS test.

3. Characterization

Nuclei magnetic resonance (NMR) spectra (^1^H NMR, ^7^Li NMR and ^19^F NMR) were recorded using Bruker DPX 300 MHz spectrometers. A scanning electron microscope (SEM, Zeiss SUPRA-55) was used for the investigation of the morphology and elemental distribution of the samples. TEM samples were prepared using the FIB, and HR-TEM was performed using a JEM-100F microscope. X-ray photoelectron spectrometry (XPS) on a Thermo Scientific Escalab 250Xi spectrometer was used to investigate the chemical states of the selected elements. *In-situ* FTIR spectra were documented by NICOLET iS50 Frontier-FTIR Spectrometer (Thermos Scientific, America).

4. DFT calculation and MD simulation

All the DFT computations were finished with the Gaussian 09 package. Four different combinations of density functional methods and basis were performed the binding energy calculation between Li^+^ and solvents, including B3LYP/6-31+G(d), B3LYP/6-311++G(d,p), B3LYP/DGDZVPS and M062X/6-311++G(d,p). The Li^+^-solvent binding energy equals the single-point energy of the Li^+^-solvent compound minus the single-point energy of the corresponding solvent and Li^+^. Basis set of B3LYP/6-311++G(d,p) with DMSO implicit solvent was used to calculate the binding energy between Li^+^ and anions. Molecular dynamics simulations were performed in the Material Studio using the universal force field (UFF). The molecular structures were initially calculated by the Gaussian 09 package. Molecular dynamics simulations under the NPT ensemble after steps of energy minimization were implemented using the Nose–Hoover thermostat at 298 K and Berendsen pressure coupling method under 1 atm for 2 ns (1.0fs time step). Later molecular dynamics simulations under the NVT ensemble after steps of energy minimization were implemented using the Nose–Hoover thermostat at 298 K for 1 ns (1.0fs time step).


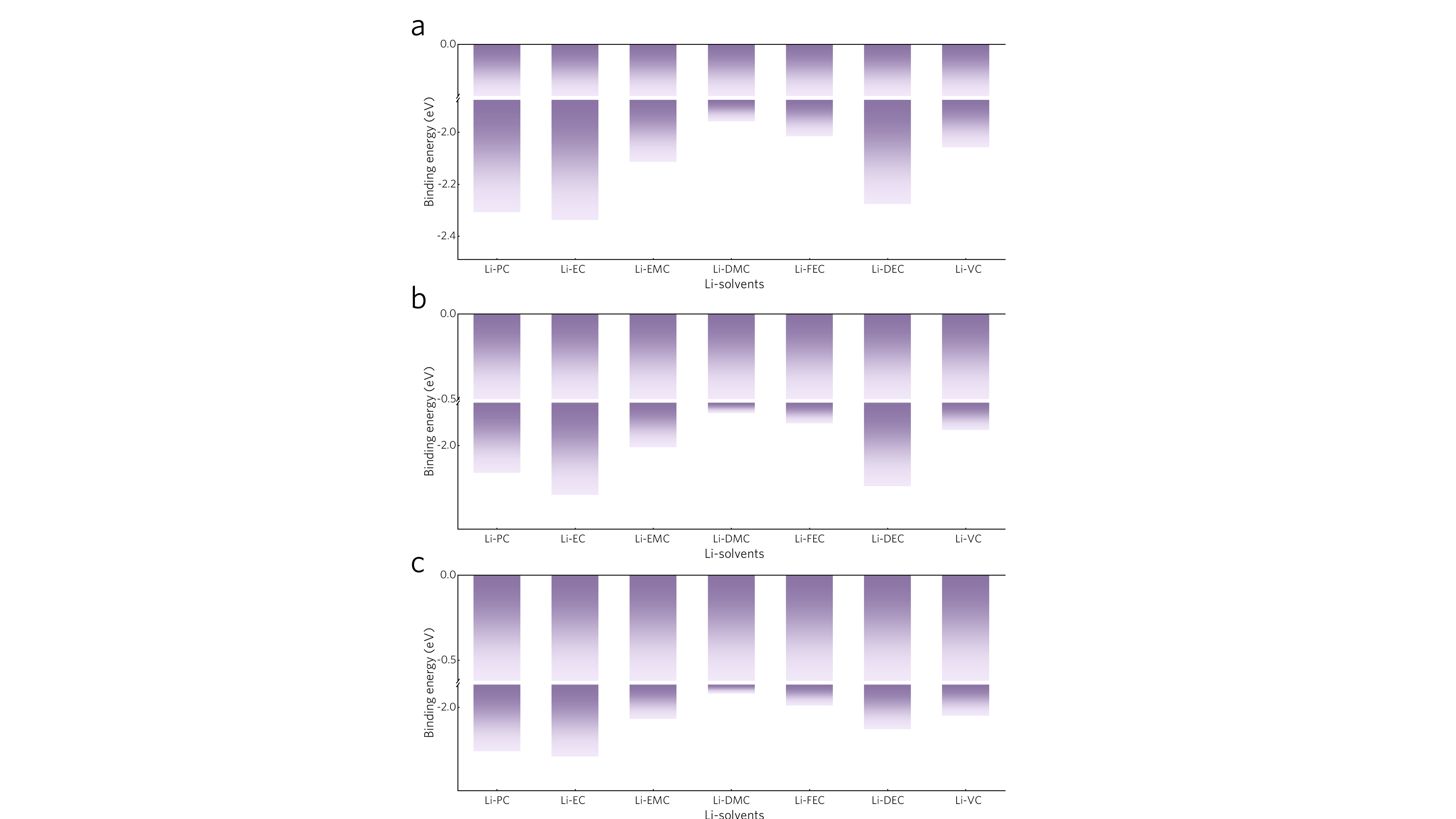


**Figure S1**. Calculated Li^+^-solvent binding energies based on the basis set of (a) B3LYP/6-311++G(d,p), (b) B3LYP/DGDZVPS and (c) M062X/6-311++G(d,p).


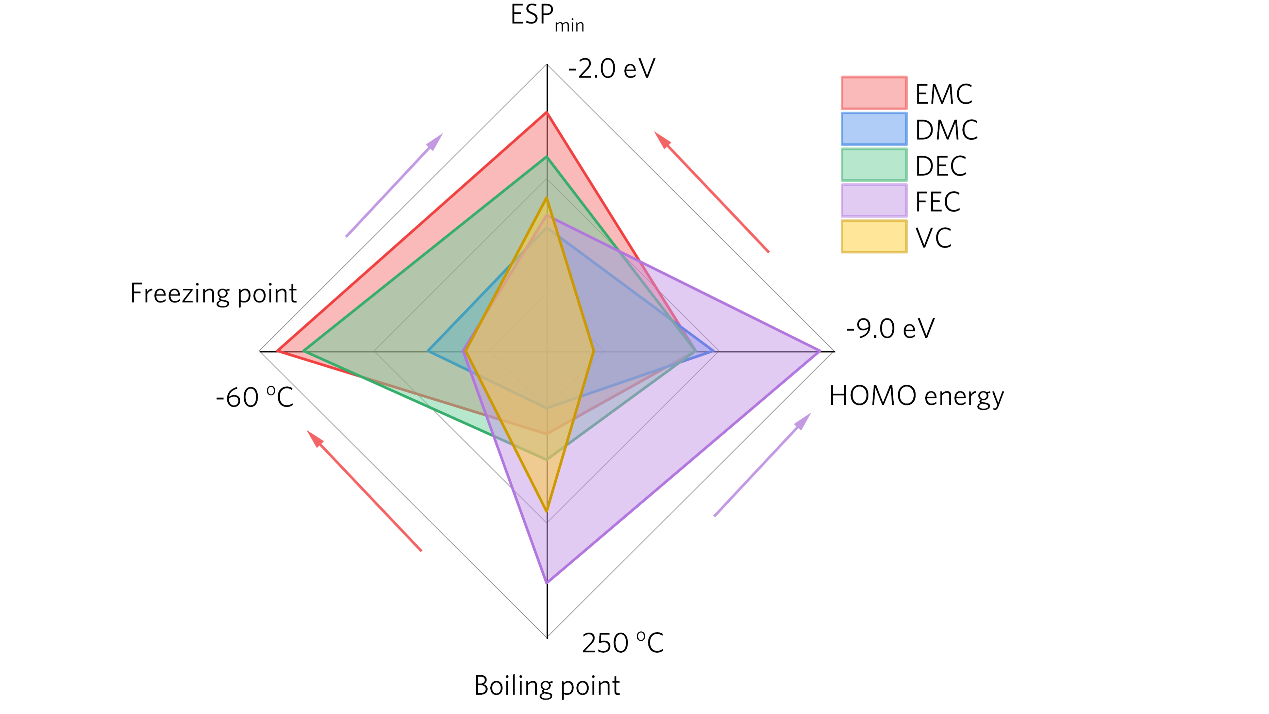


**Figure S2.** Comparison of the physicochemical properties of different carbonate solvents.


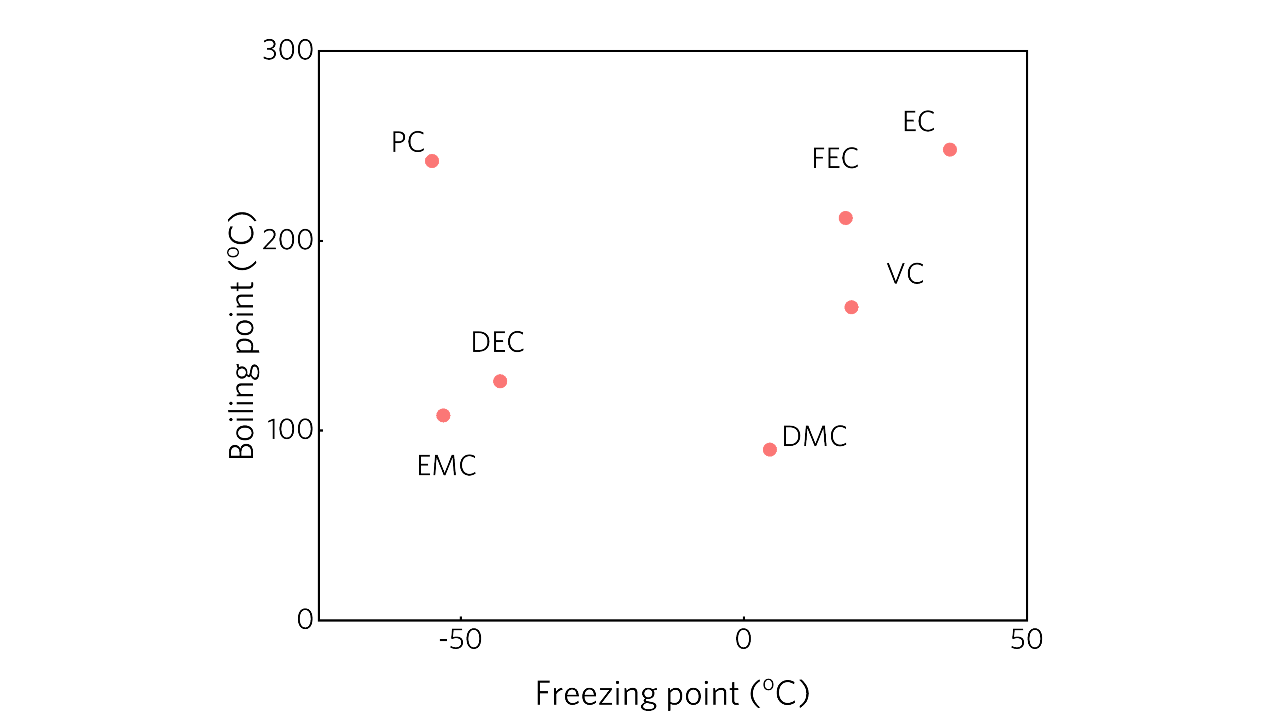


**Figure S3.** Freezing point and boiling point of different carbonate solvents.


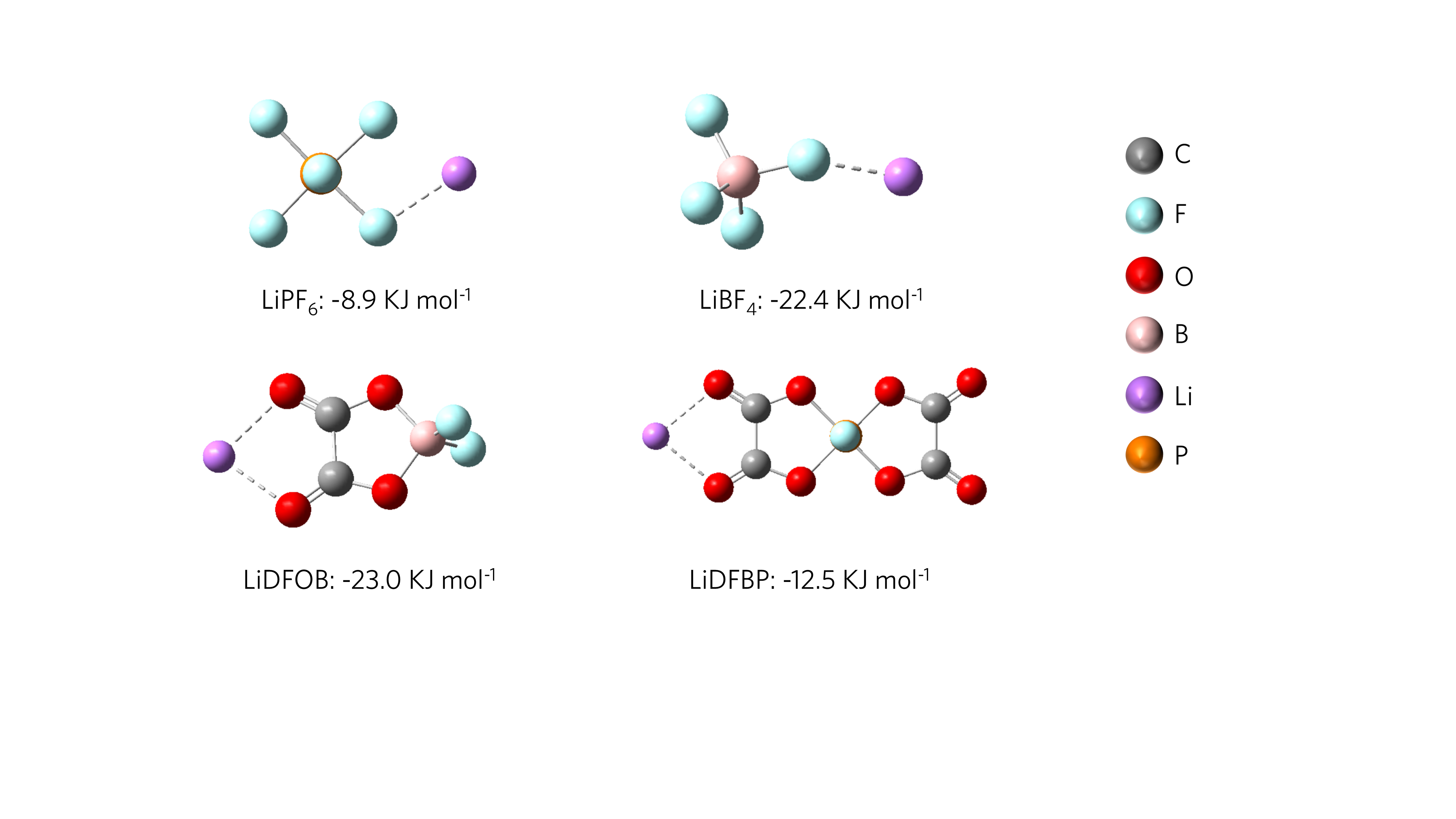


**Figure S4**. Binding energies between Li^+^ and different anions. The basis set is B3LYP/6-311++G(d,p) with DMSO implicit solvent for single-point energy calculation.


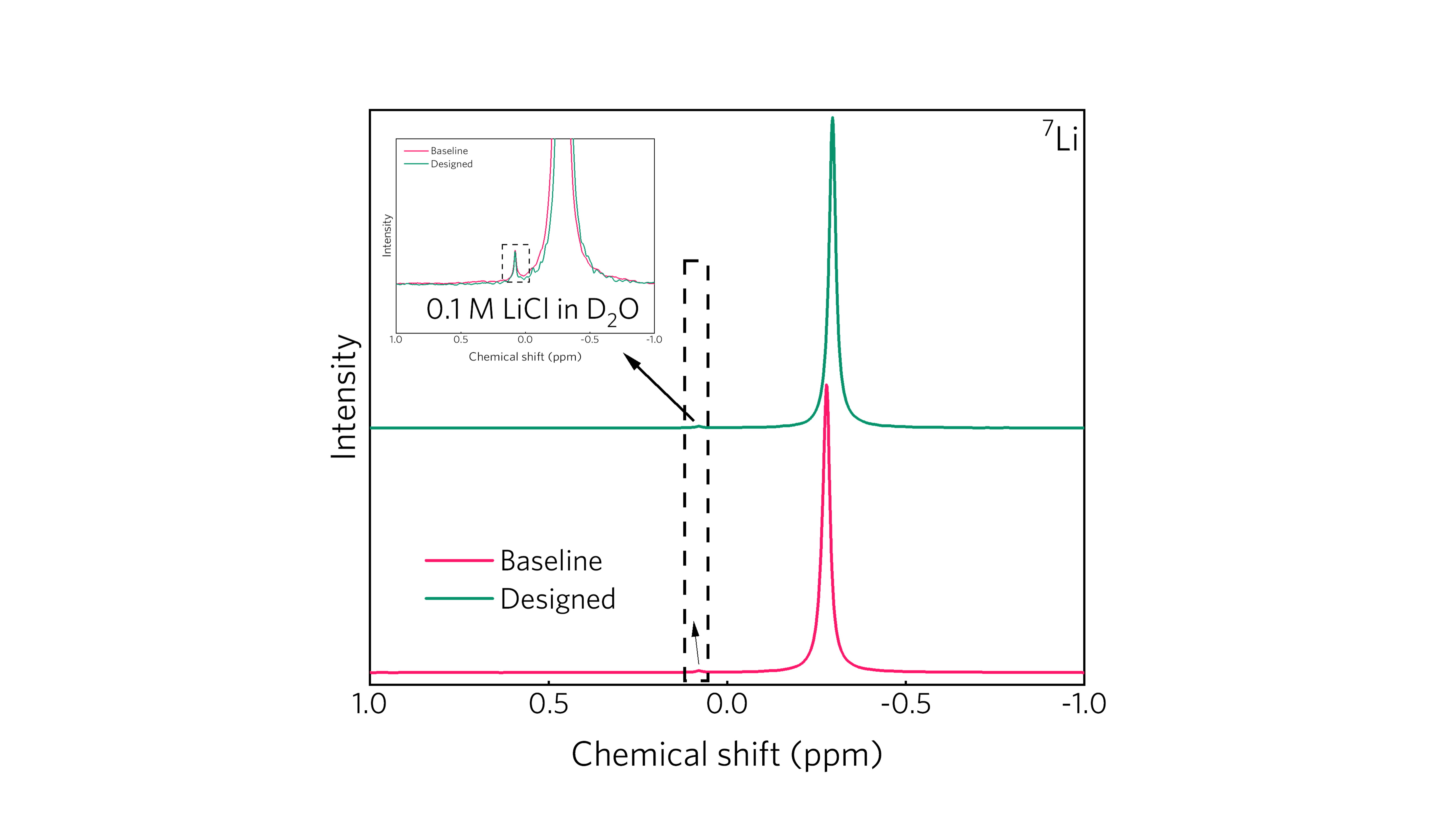


**Figure S5**. ^7^Li NMR spectra of different electrolytes.


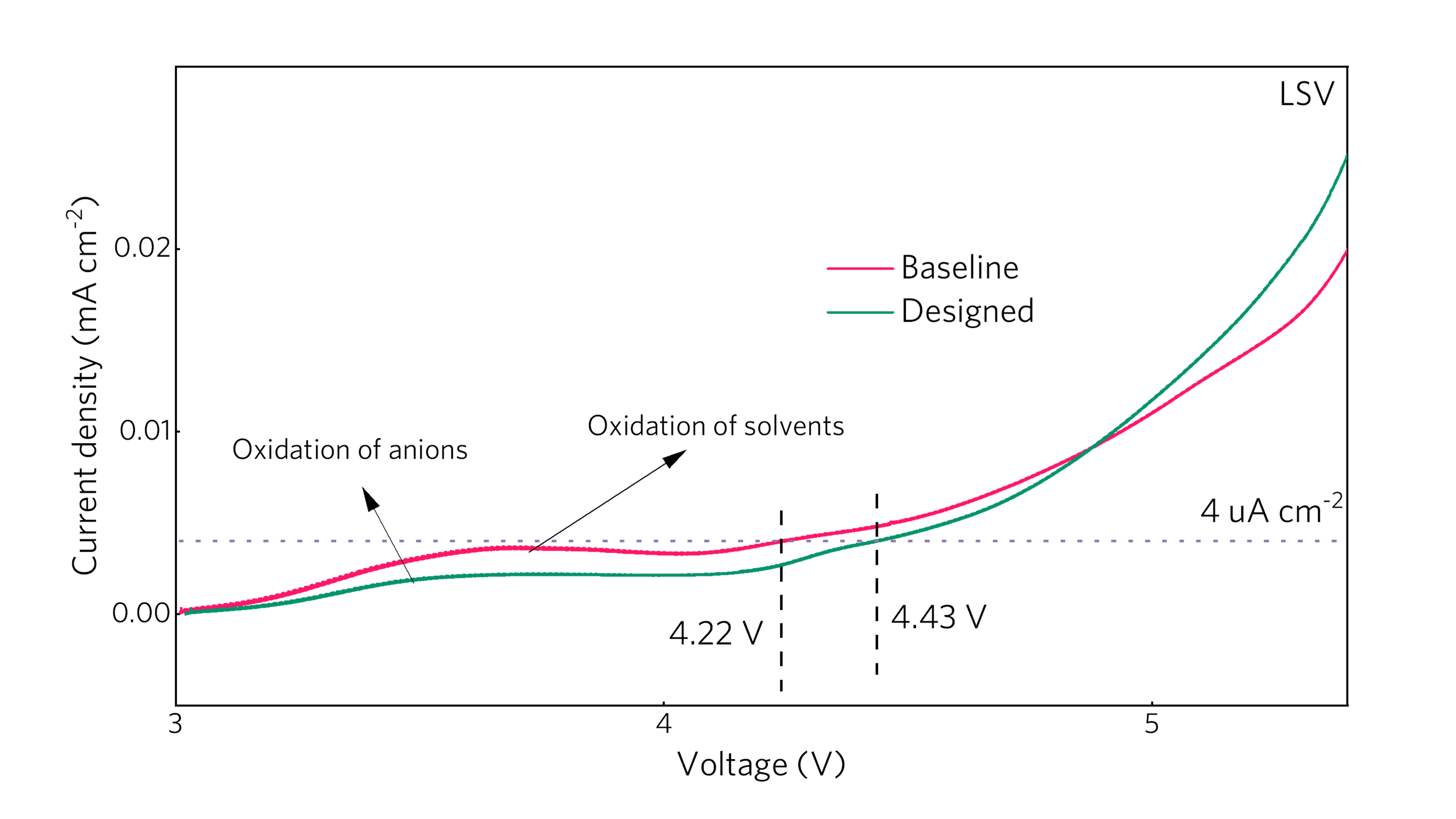


**Figure S6.** LSV curves of the baseline electrolyte and designed electrolytes.


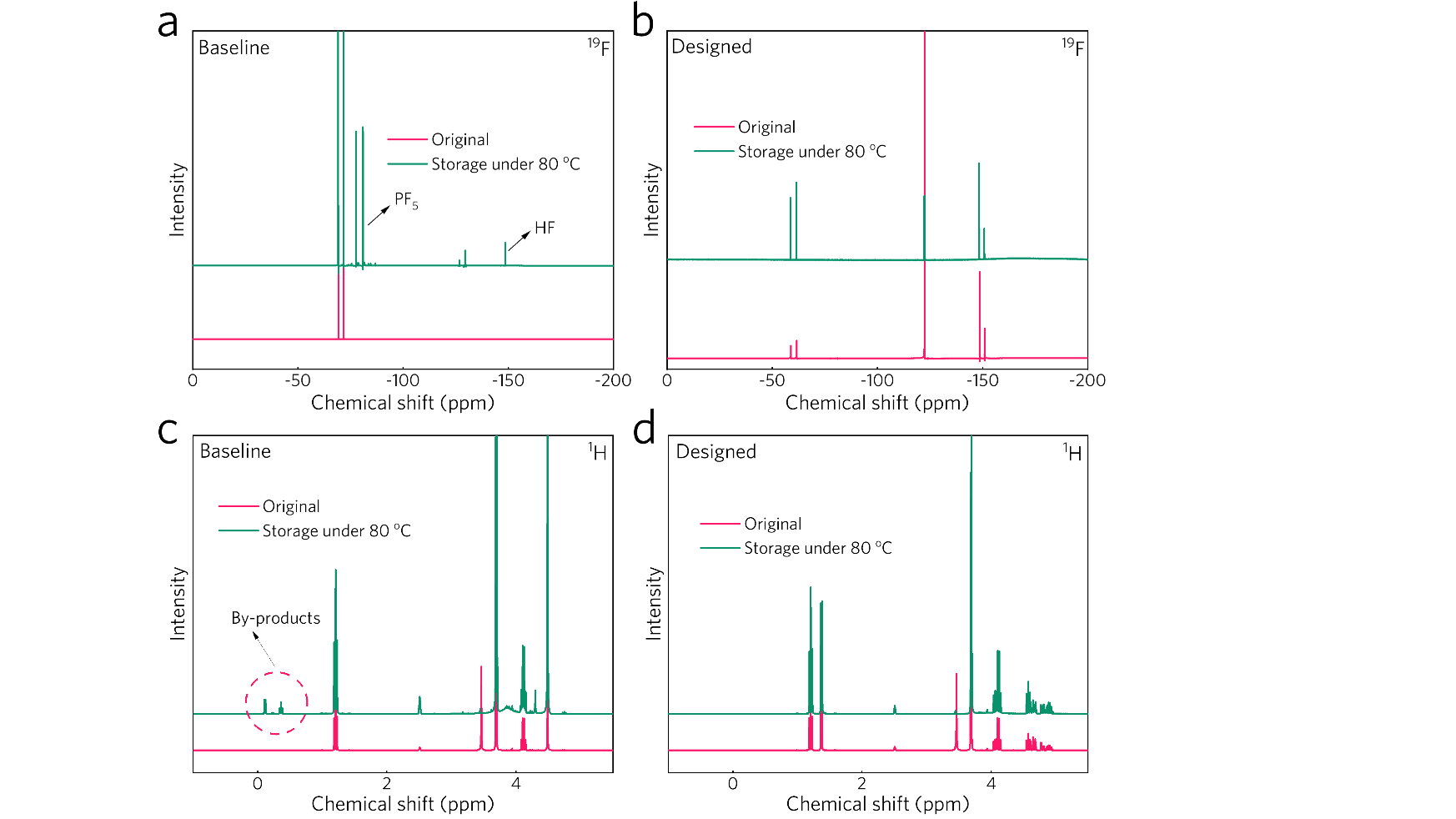


**Figure S7.** ^19^F NMR spectra of the (a) baseline electrolyte and (b) designed electrolyte before and after 144 hours of storage under 80 ^o^C. ^1^H NMR spectra of the (c) baseline electrolyte and (d) designed electrolyte before and after 144 hours of storage under 80 ^o^C.


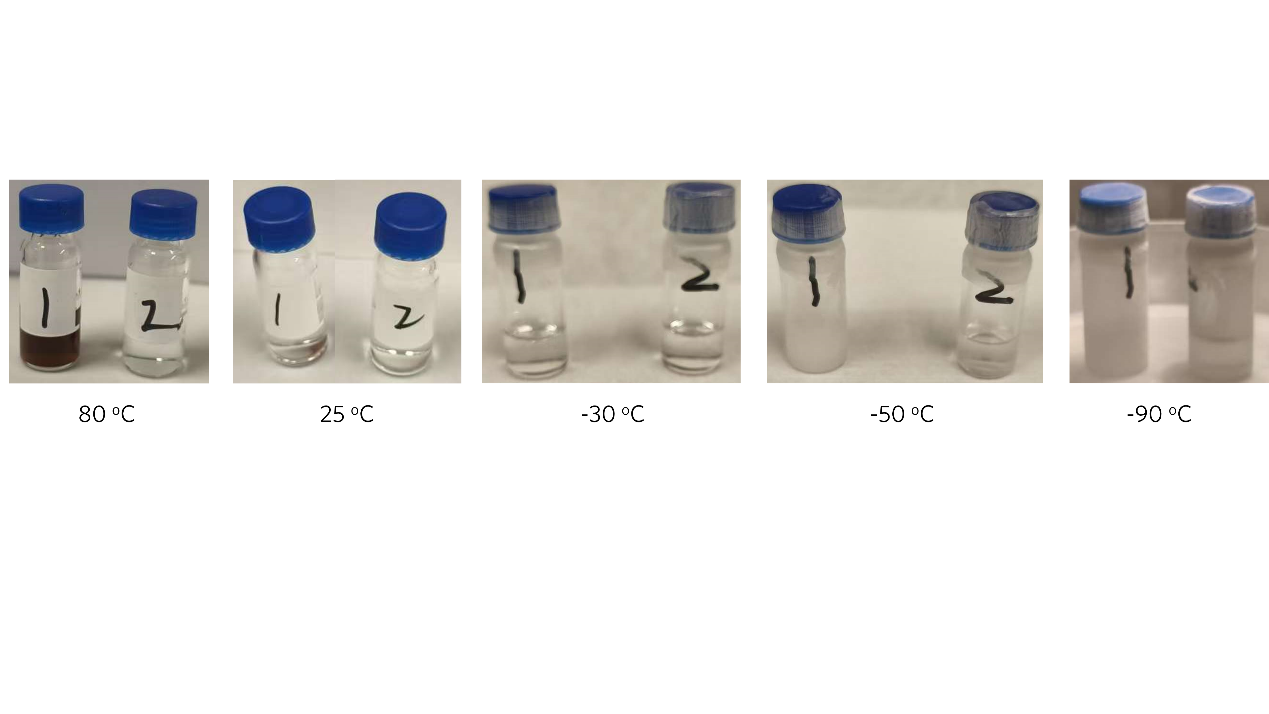


**Figure S8.** Optical images of the baseline electrolyte and designed electrolyte under different temperatures, in which 1 and 2 refer to the baseline electrolyte and designed electrolyte, respectively.


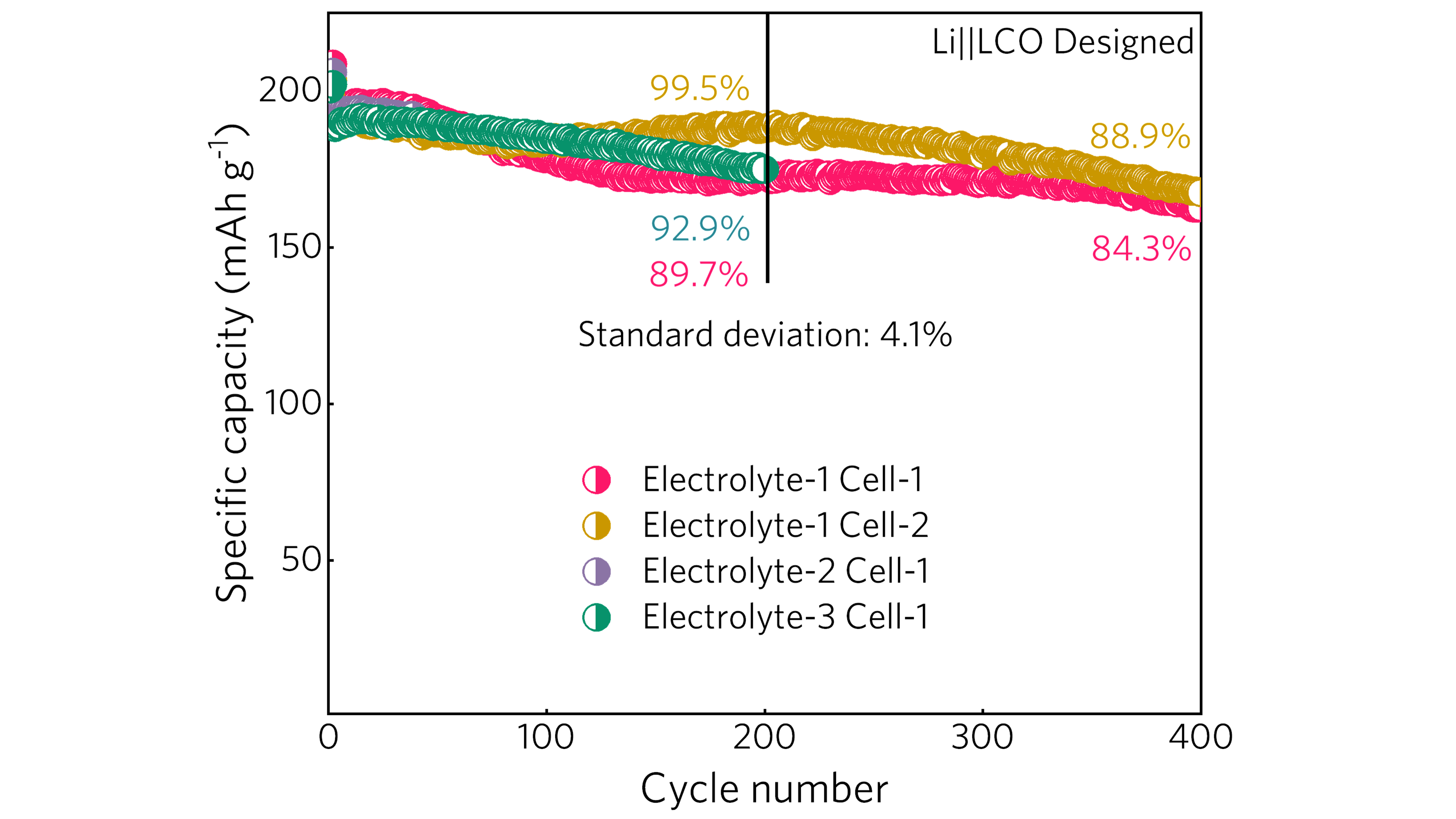


**Figure S9**. Cycling performance of different Li||LCO half-cells using different batches of electrolyte.


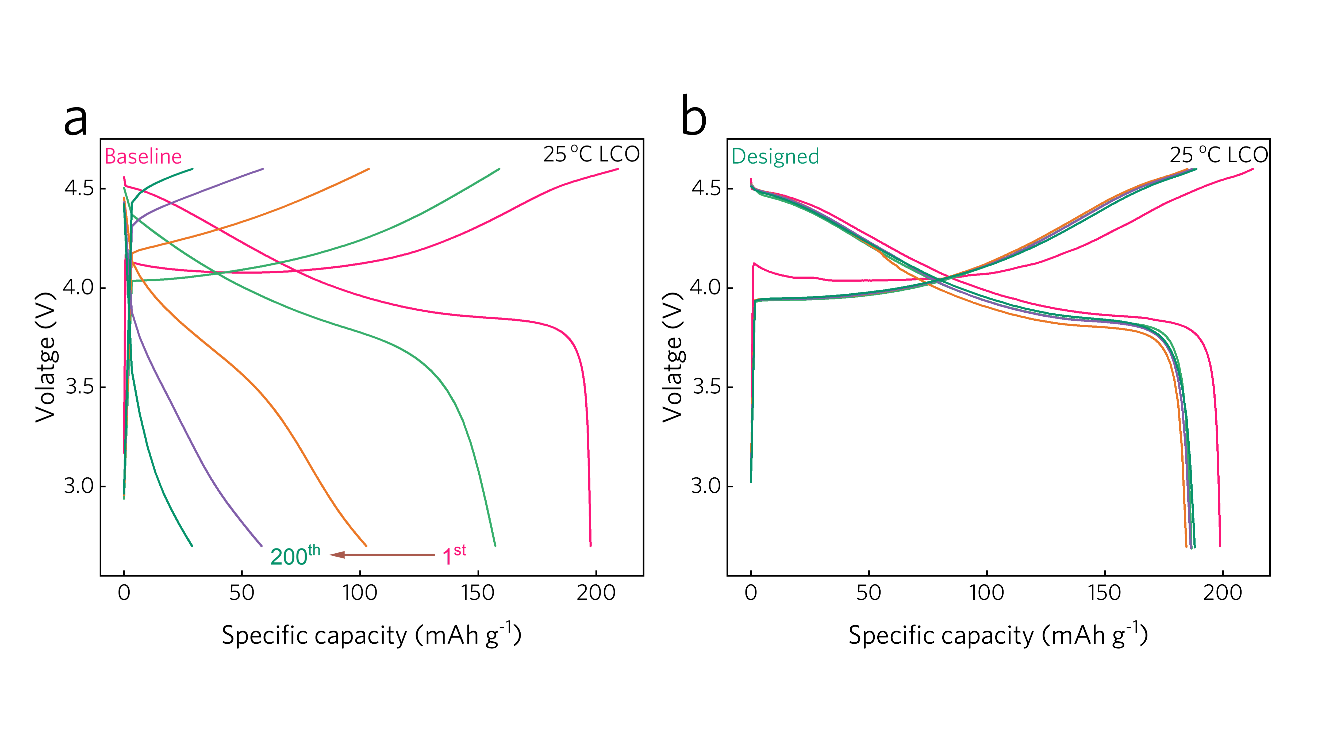


**Figure S10.** Charging and discharging profiles of the Li||LCO half-cells using (a) baseline electrolyte and (b) designed electrolyte at 25 ^o^C.


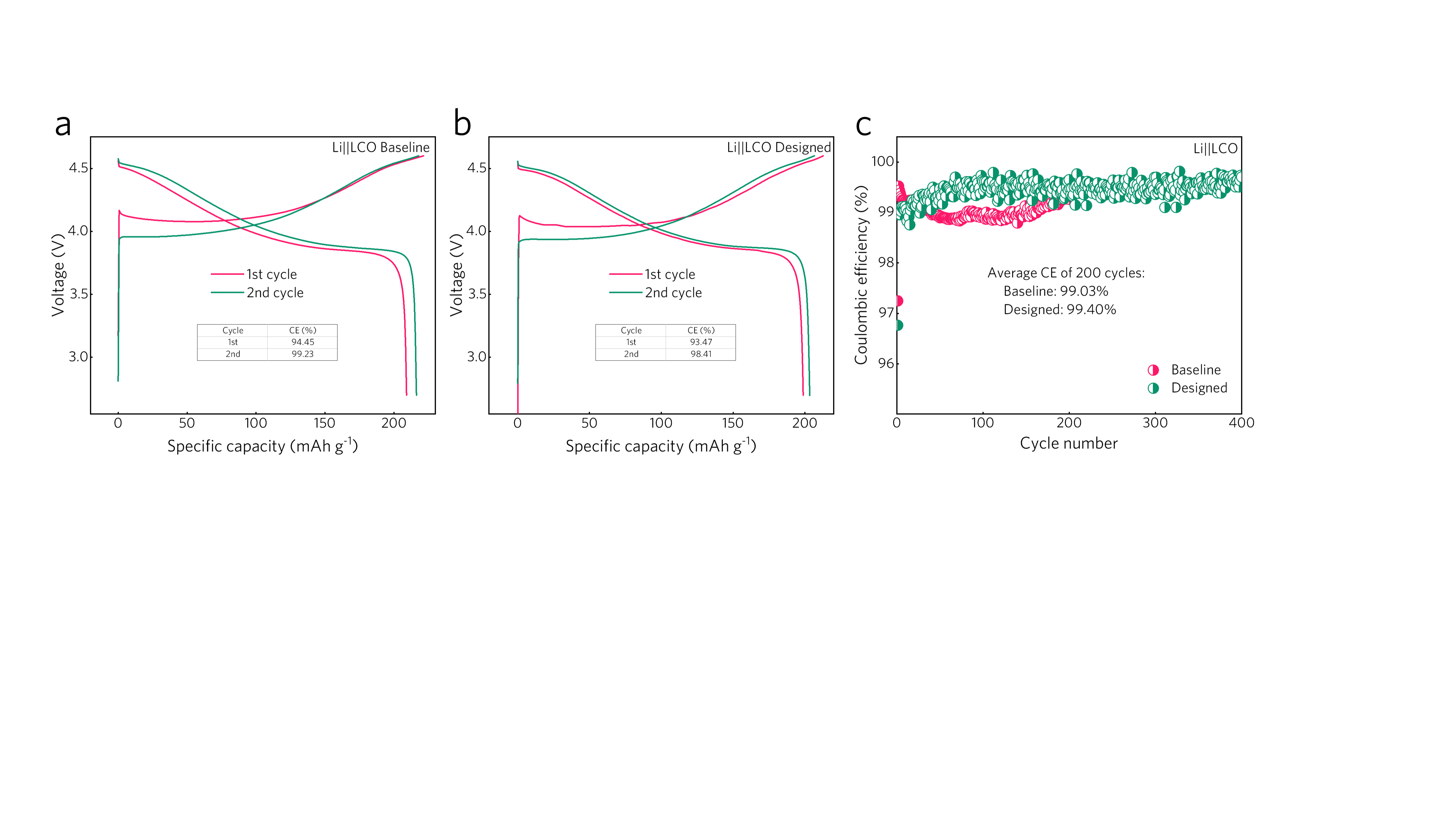


**Figure S11**. Charge/discharge profiles and CE of the activation cycles for Li||LCO half-cells using (a) baseline and (b) designed electrolyte. (c) CE during long-term cycling of Li||LCO half-cells using different electrolytes and the calculated average CE after 200 cycles. The average CE of designed electrolyte after 400 cycles is 99.45%.


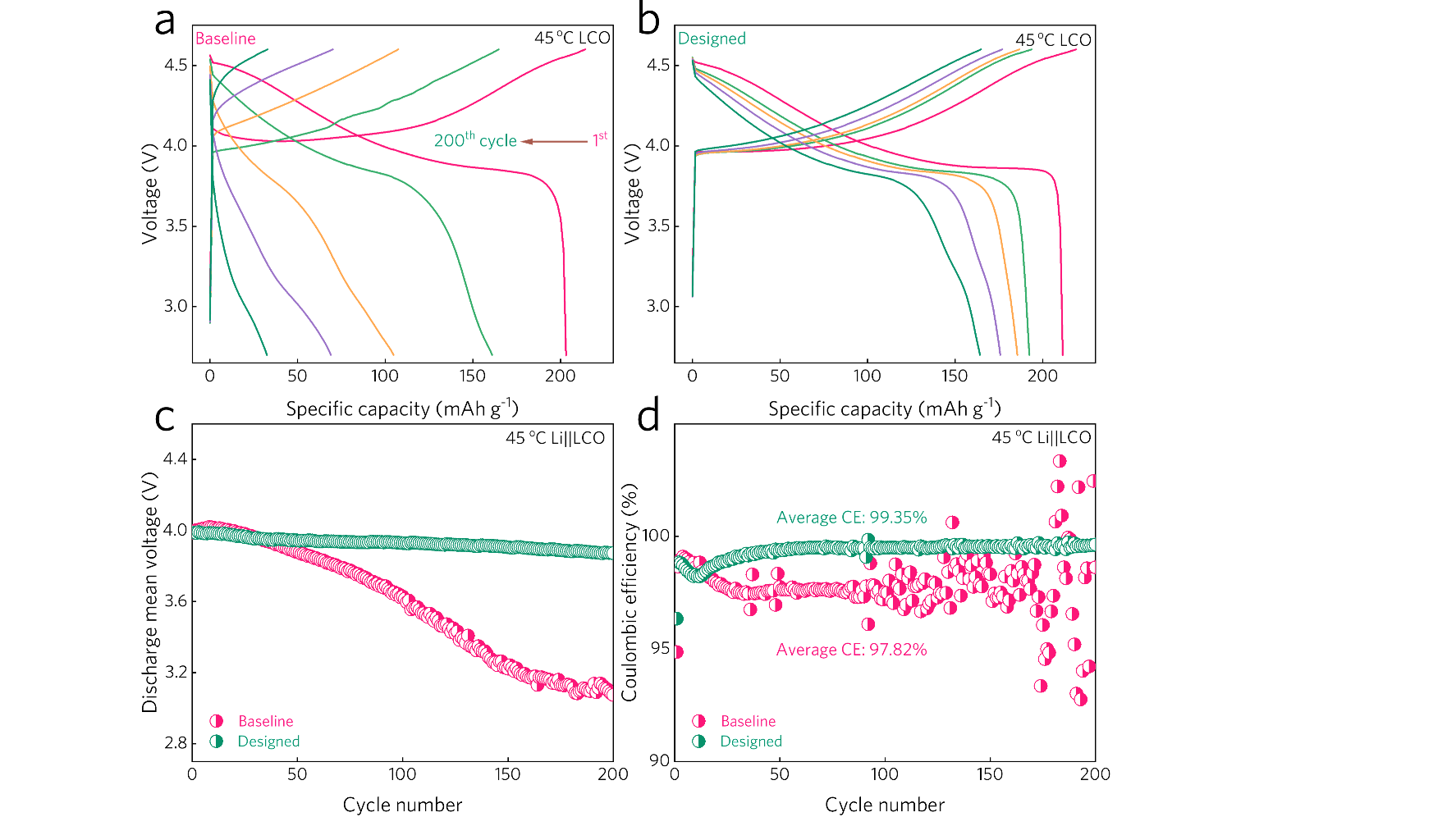


**Figure S12.** Charging and discharging profiles of the Li||LCO half-cells using (a) baseline electrolyte and (b) designed electrolyte at 45 ^o^C. (c) Mean voltage upon discharging and (d) Coulombic efficiency of the Li||LCO half-cells using different electrolytes at 45 ^o^C.


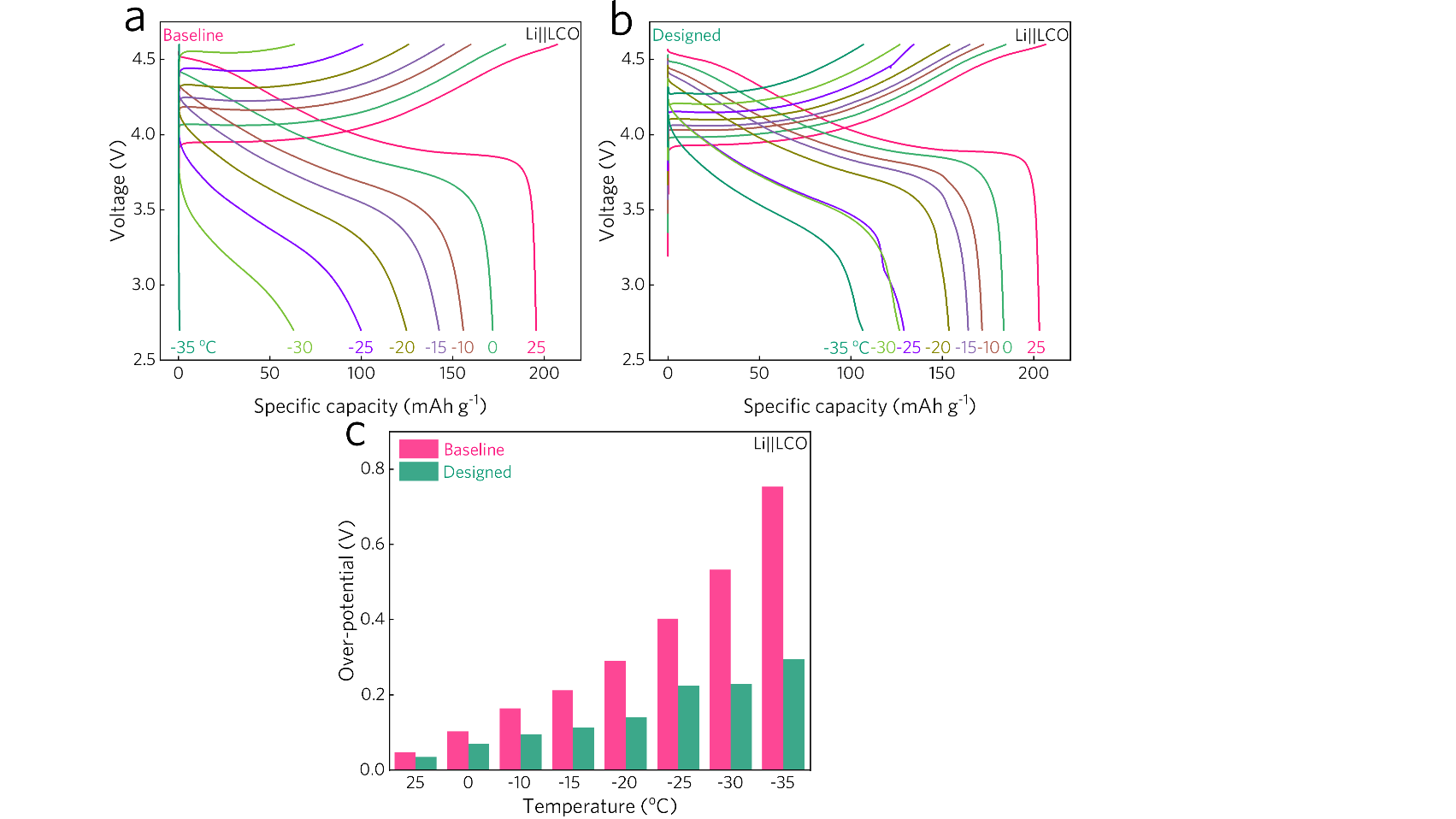


**Figure S13.** Charge/discharge profiles of the Li||LCO half-cells using (a) baseline electrolyte and (b) designed electrolyte at different temperatures. (c) Over-potential of the Li||LCO half-cells using different electrolytes at different temperatures.


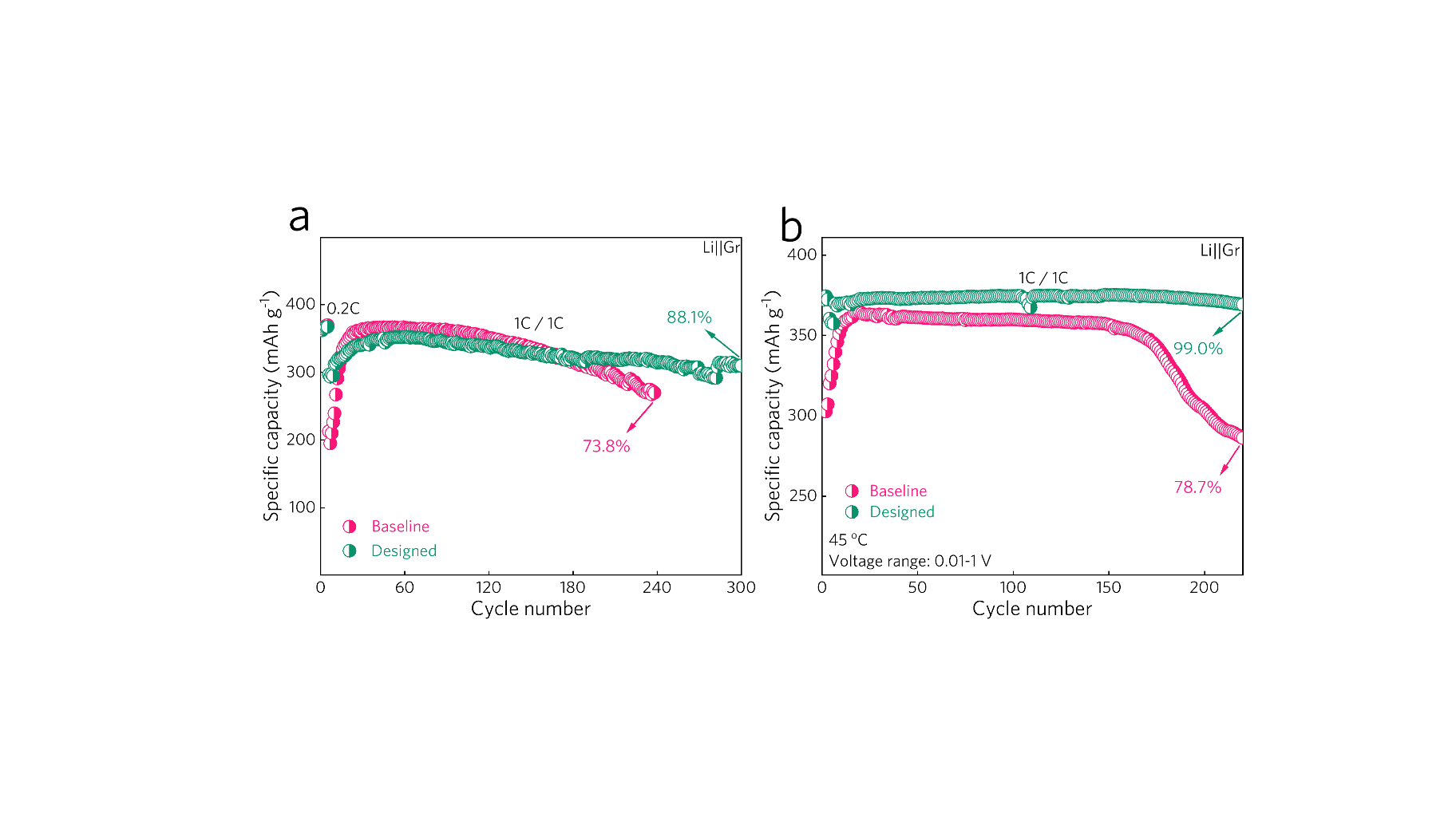


**Figure S14.** Long-term cycling of the Li||graphite half-cells using different electrolytes at (a) 25 ^o^C and (b) 45 ^o^C.


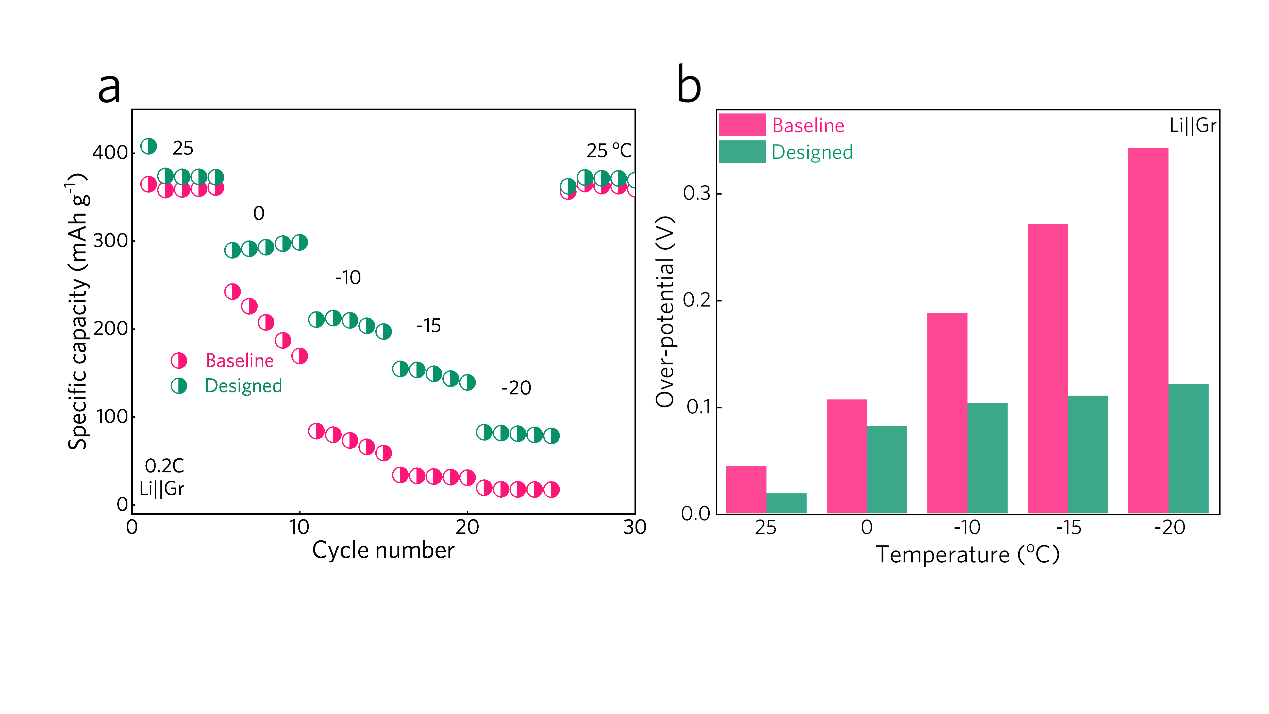


**Figure S15.** (a) Capacity retention and (b) over-potential of the Li||graphite half-cells using different electrolytes at different temperatures.


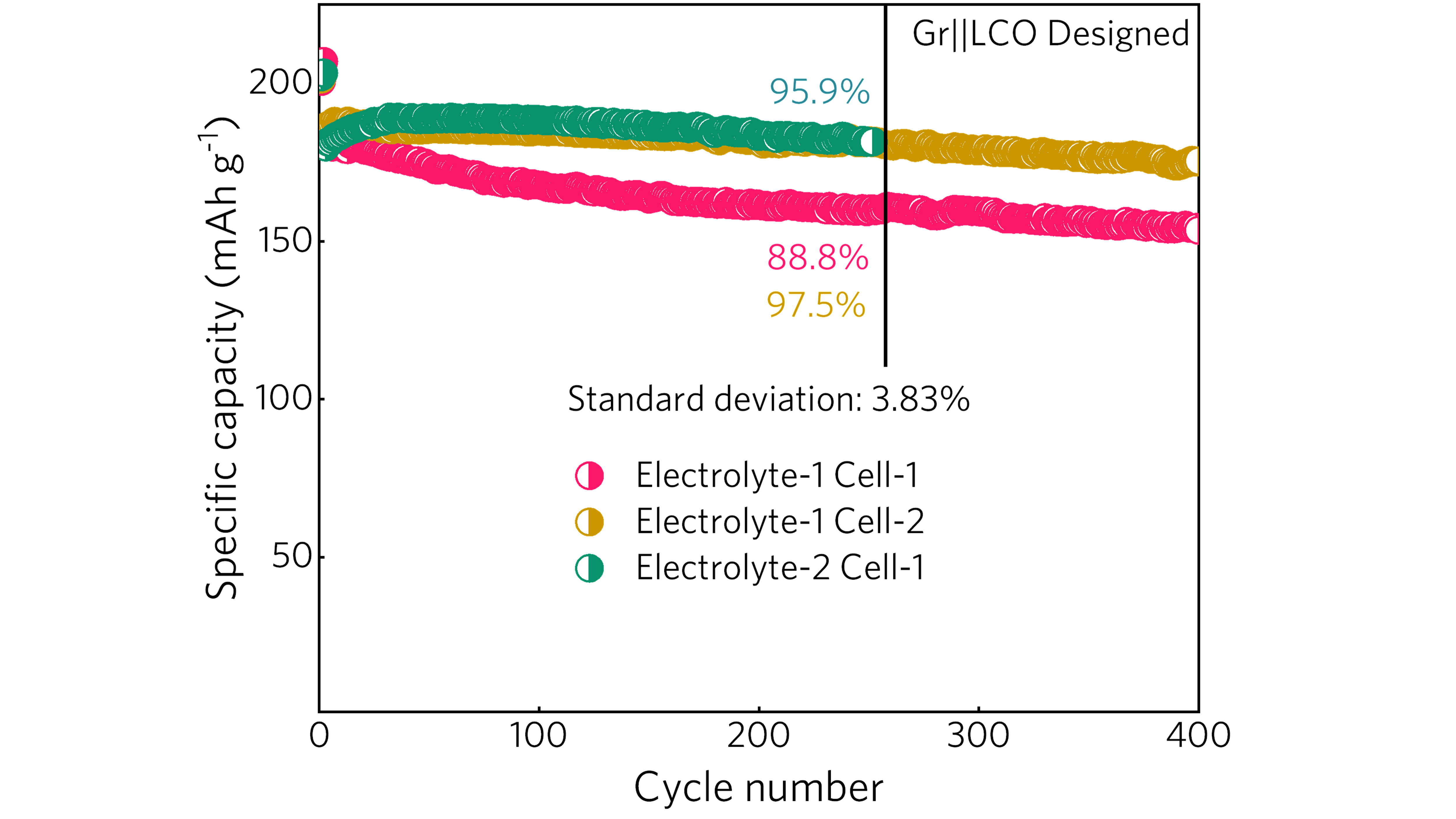


**Figure S16**. Cycling performance of graphite||LCO full-cells using different batches of electrolyte.


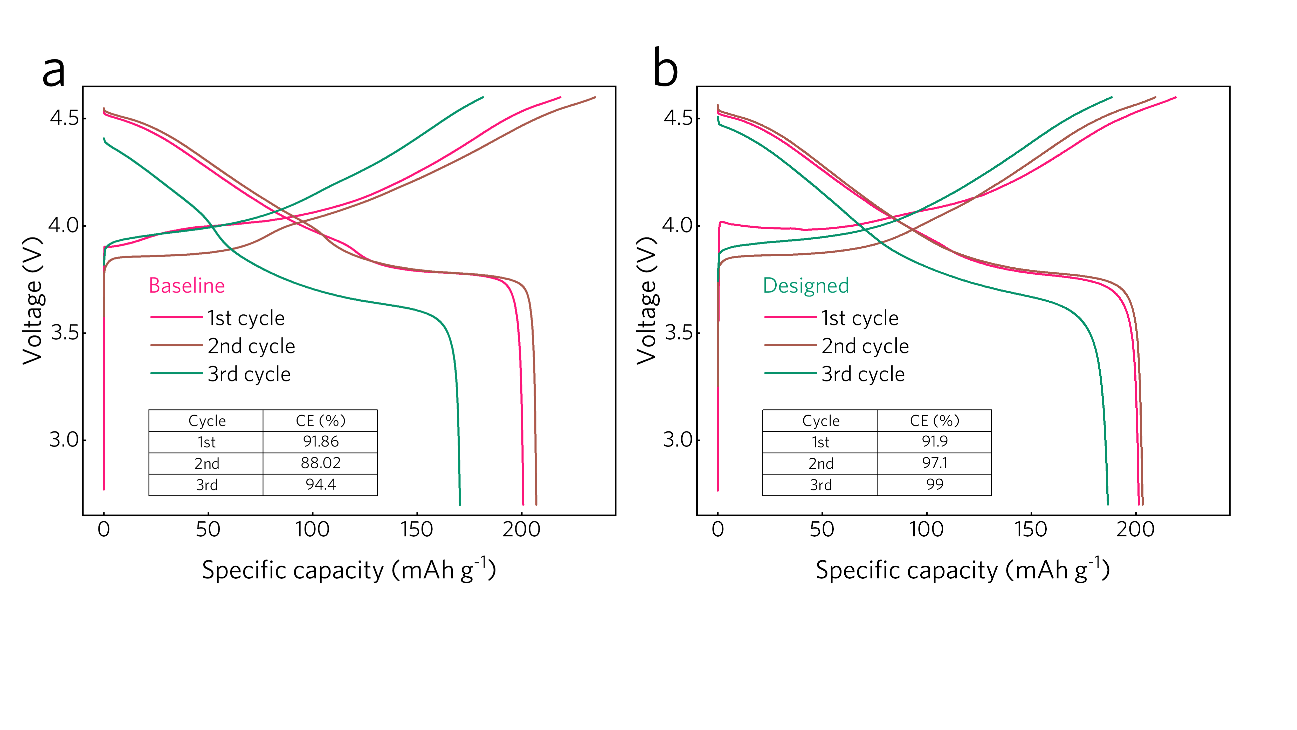


**Figure S17.** Charge/discharge profiles of the graphite||LCO full-cells using (a) baseline electrolyte and (b) designed electrolyte.


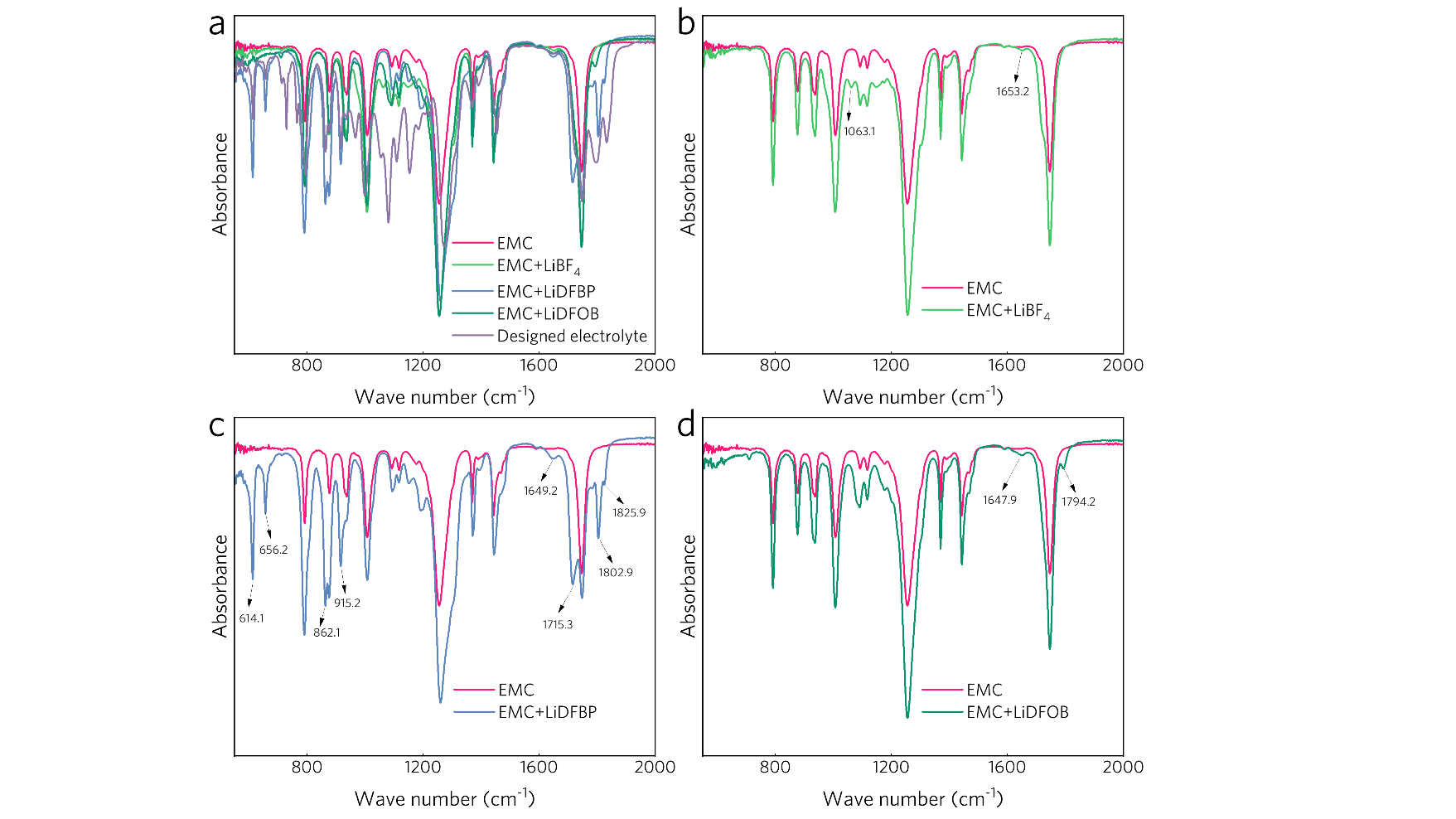


**Figure S18.** (a) FTIR spectra of EMC solvent, EMC solution with different lithium salts and the designed electrolyte. FTIR of EMC solvent and the EMC solution with 20 wt.% (b) LiBF_4_, (c) LiDFBP and (d) LiDFOB.


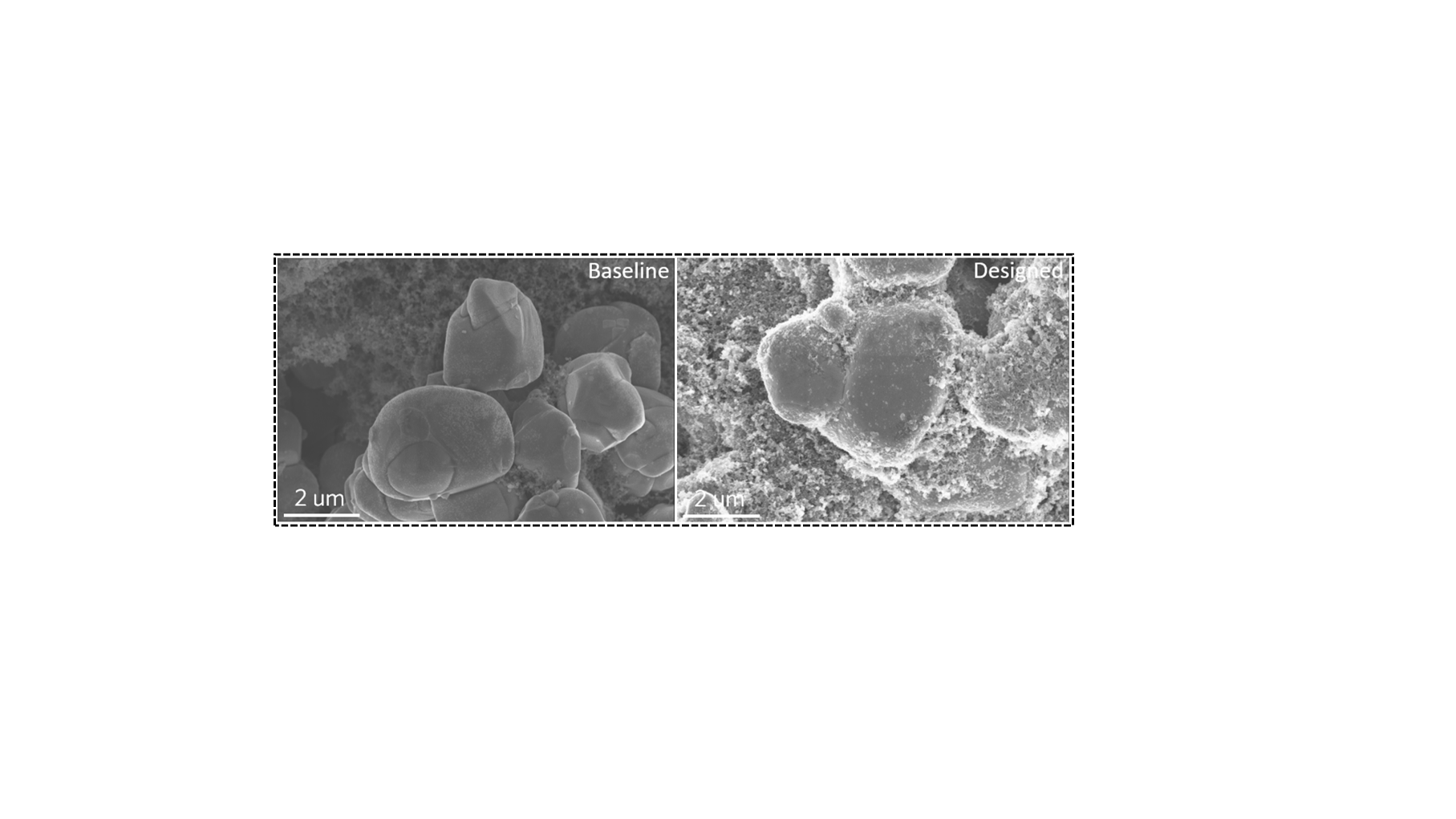


**Figure S19.** SEM images of the LCO after 2 activation cycles with a current density at 0.2 C (1 C=200 mAh g^-1^) using different electrolytes.


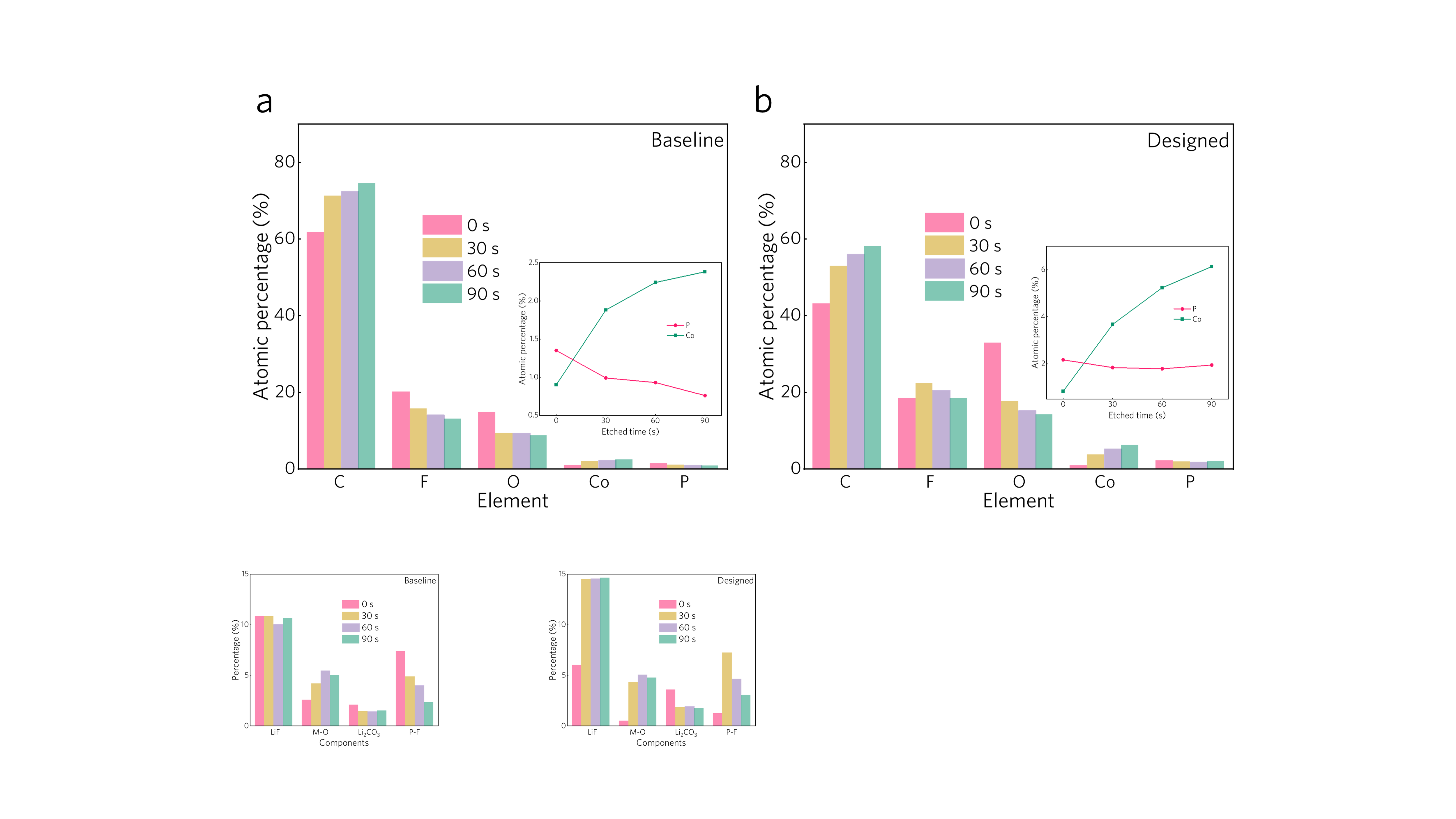


**Figure S20**. Atomic percentages of different elements on LCO surface after 50 cycles in (a) baseline electrolyte and (b) designed electrolyte with different etching times.


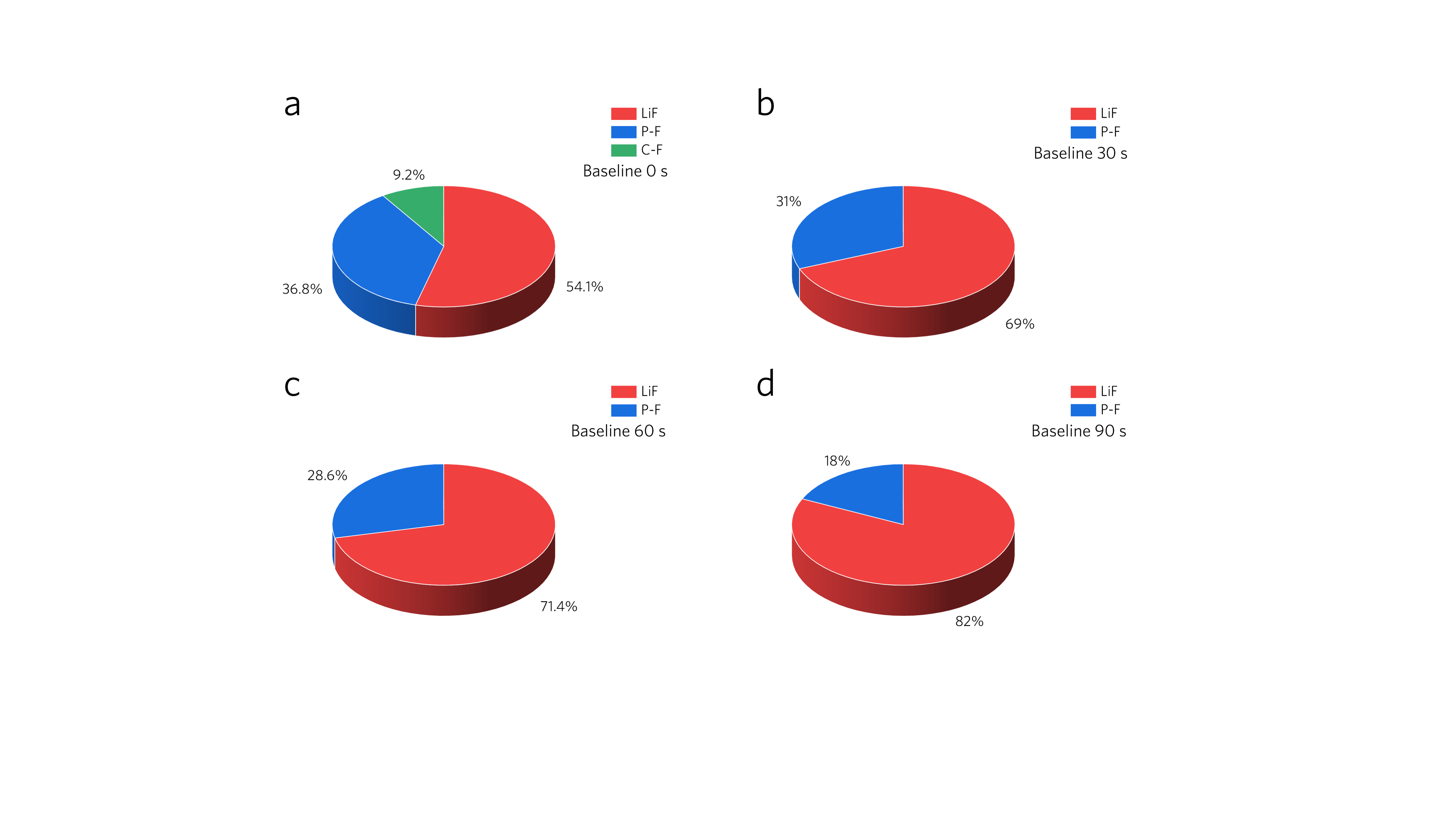


**Figure S21**. Percentages of the fitted areas for different components based on F1s XPS spectra with (a) 0, (b) 30, (c) 60 and (d) 90 seconds etching on the LCO surface after 50 cycles in the baseline electrolyte.


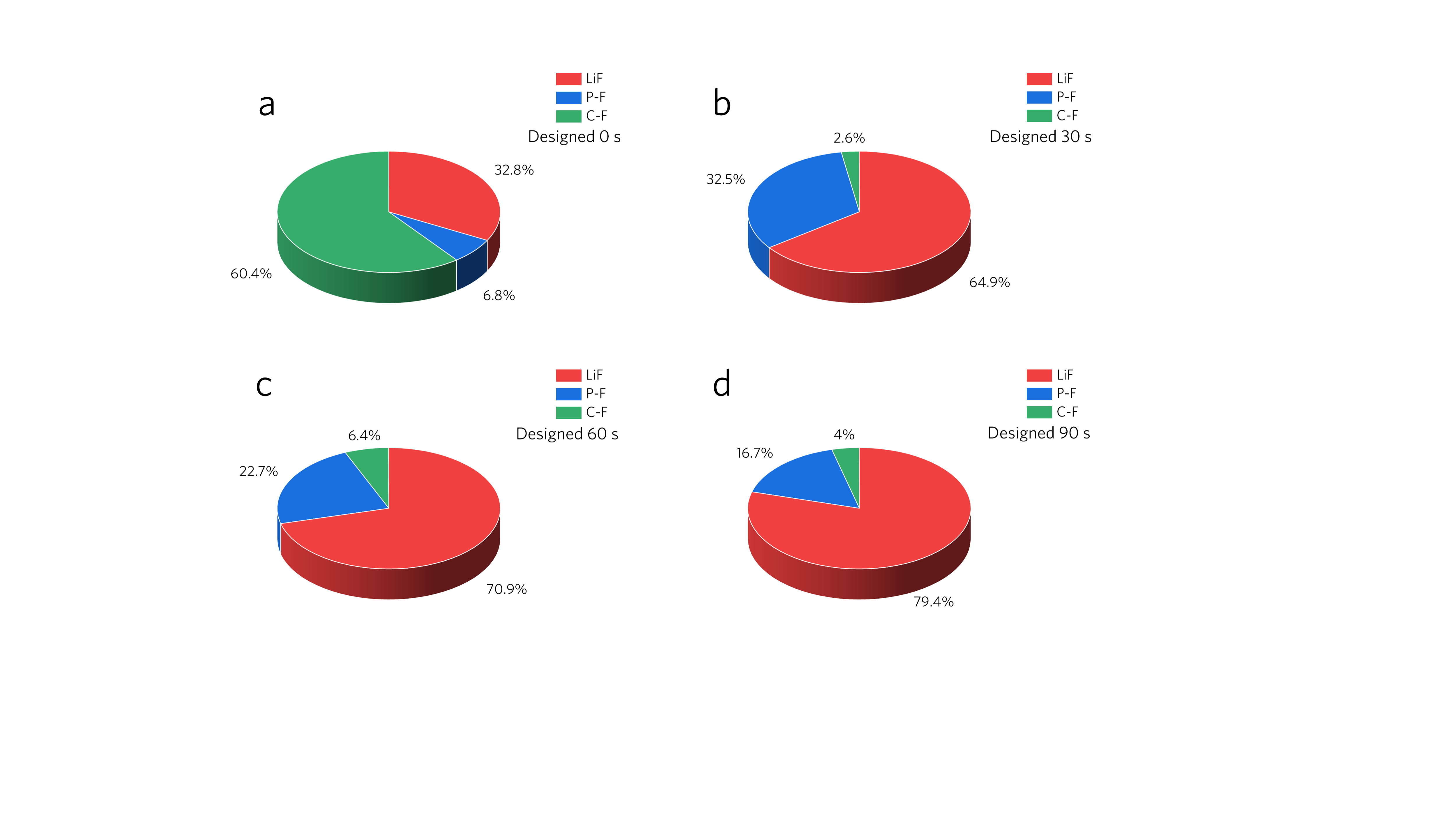


**Figure S22**. Percentages of the fitted areas for different components based on F1s XPS spectra with (a) 0, (b) 30, (c) 60 and (d) 90 seconds etching on the LCO surface after 50 cycles in the designed electrolyte.


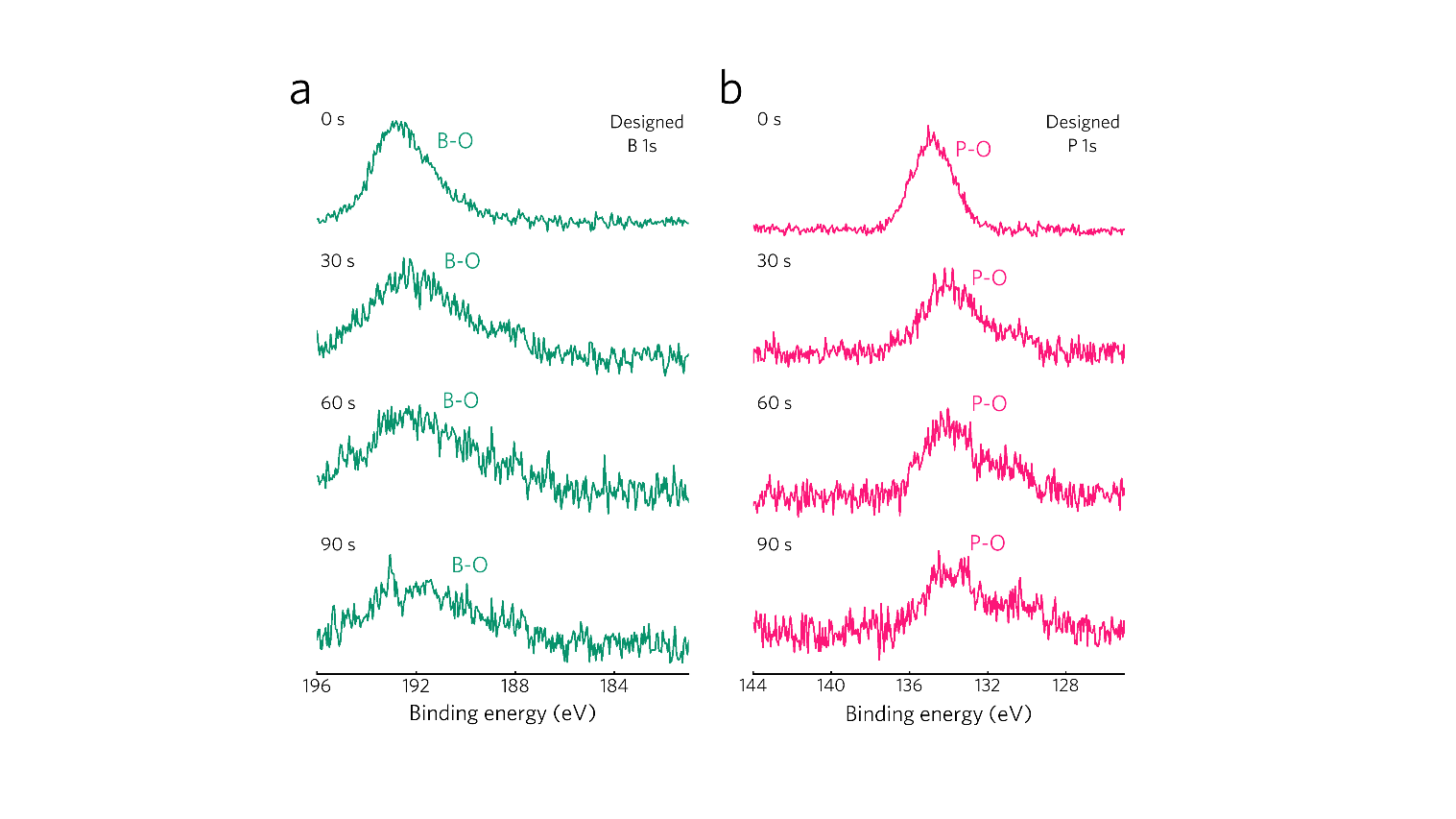


**Figure S23.** (a) B1s and (b) P1s XPS core spectra of the LCO after 50 cycles upon different etching times.


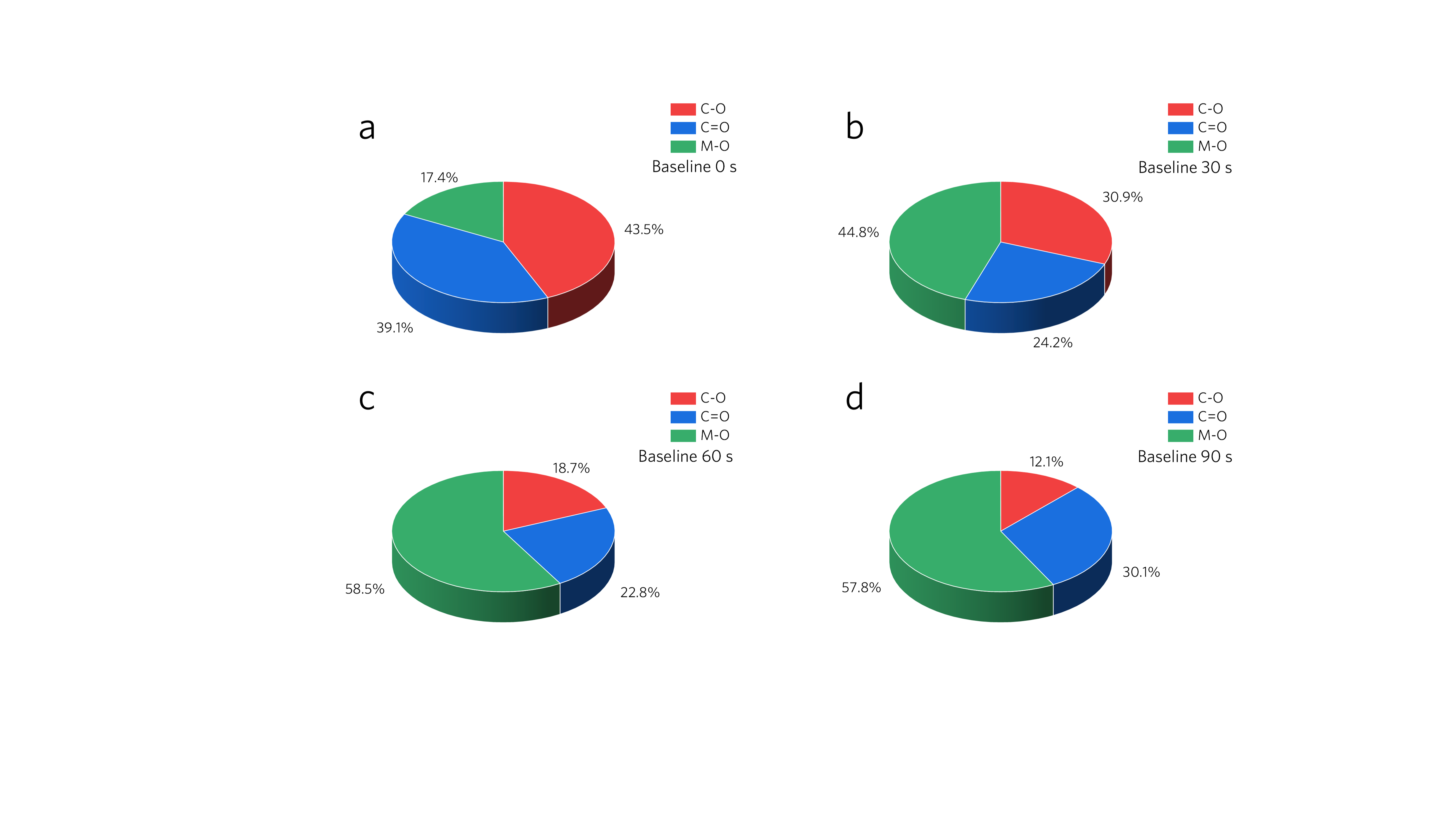


**Figure S24**. Percentages of the fitted areas for different components based on O1s XPS spectra with (a) 0, (b) 30, (c) 60 and (d) 90 seconds etching on the LCO surface after 50 cycles in the baseline electrolyte.


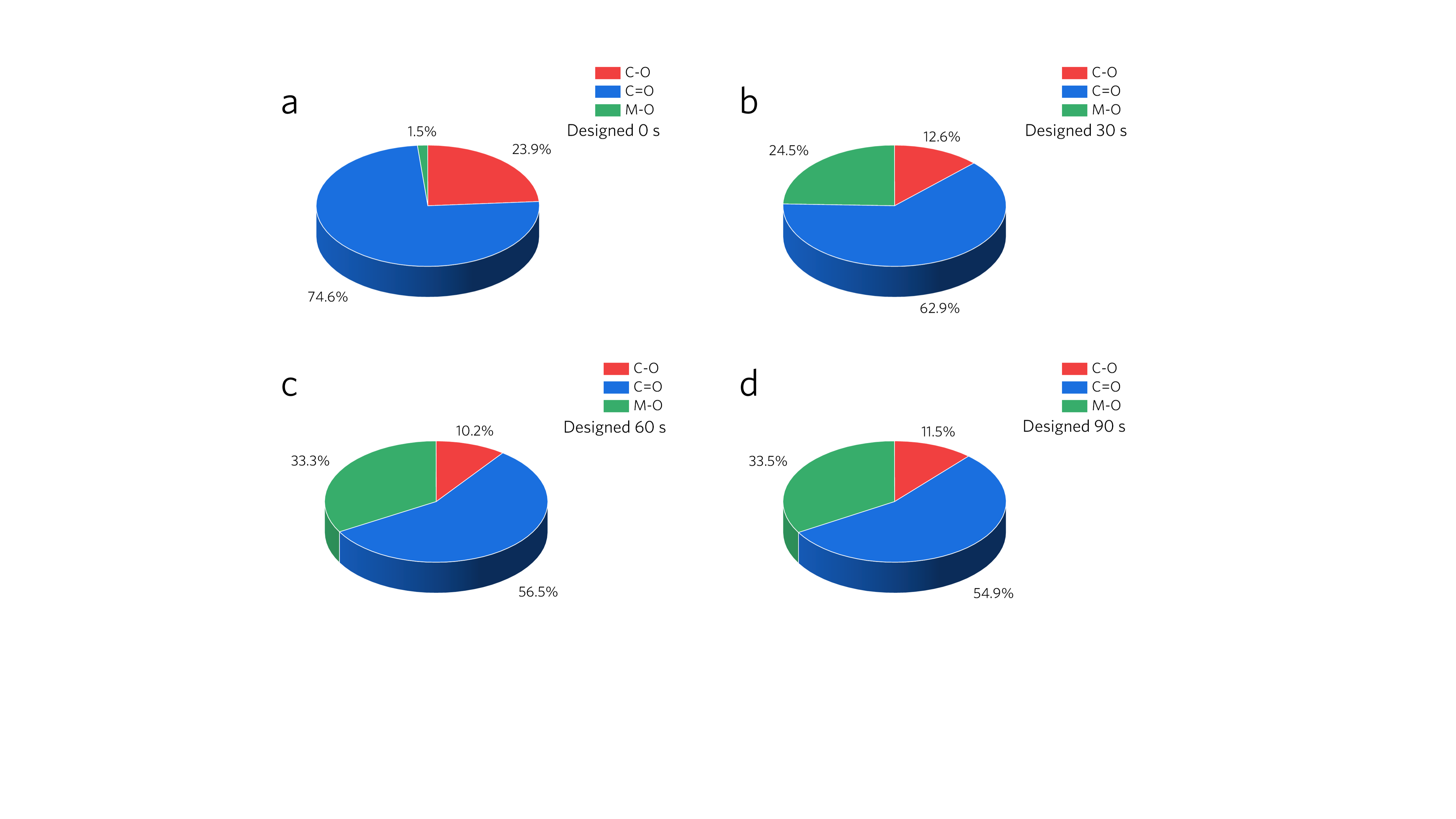


**Figure S25**. Percentages of the fitted areas for different components based on O1s XPS spectra with (a) 0, (b) 30, (c) 60 and (d) 90 seconds etching on the LCO surface after 50 cycles in the designed electrolyte.


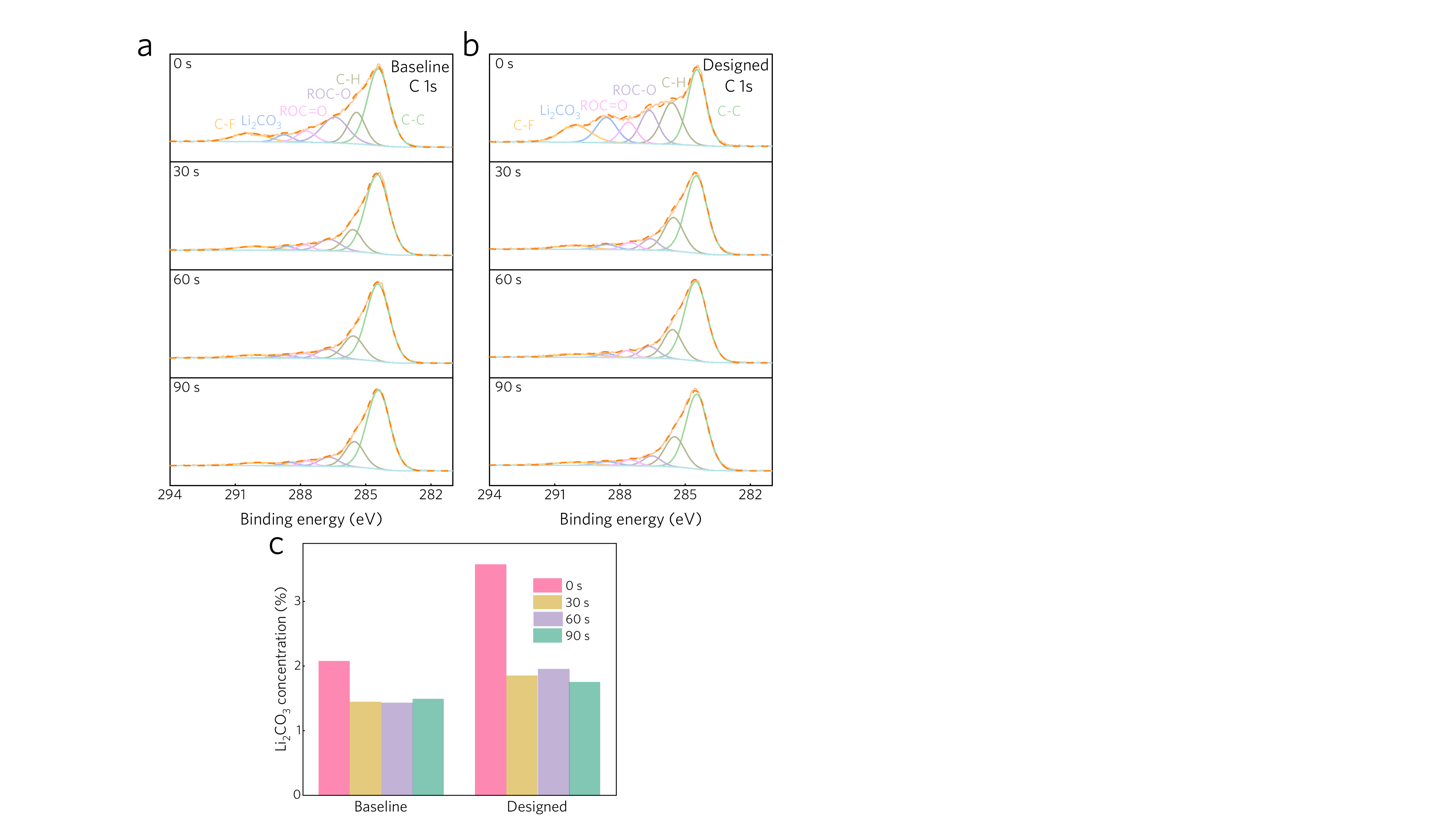


**Figure S26**. (a,b) C *1s* spectra of LCO surface after 50 cycles in (a) baseline electrolyte and (b) designed electrolyte with different etching times. (c) The concentration of Li_2_CO_3_ for the LCO surface after 50 cycles in different electrolytes with different etching times.


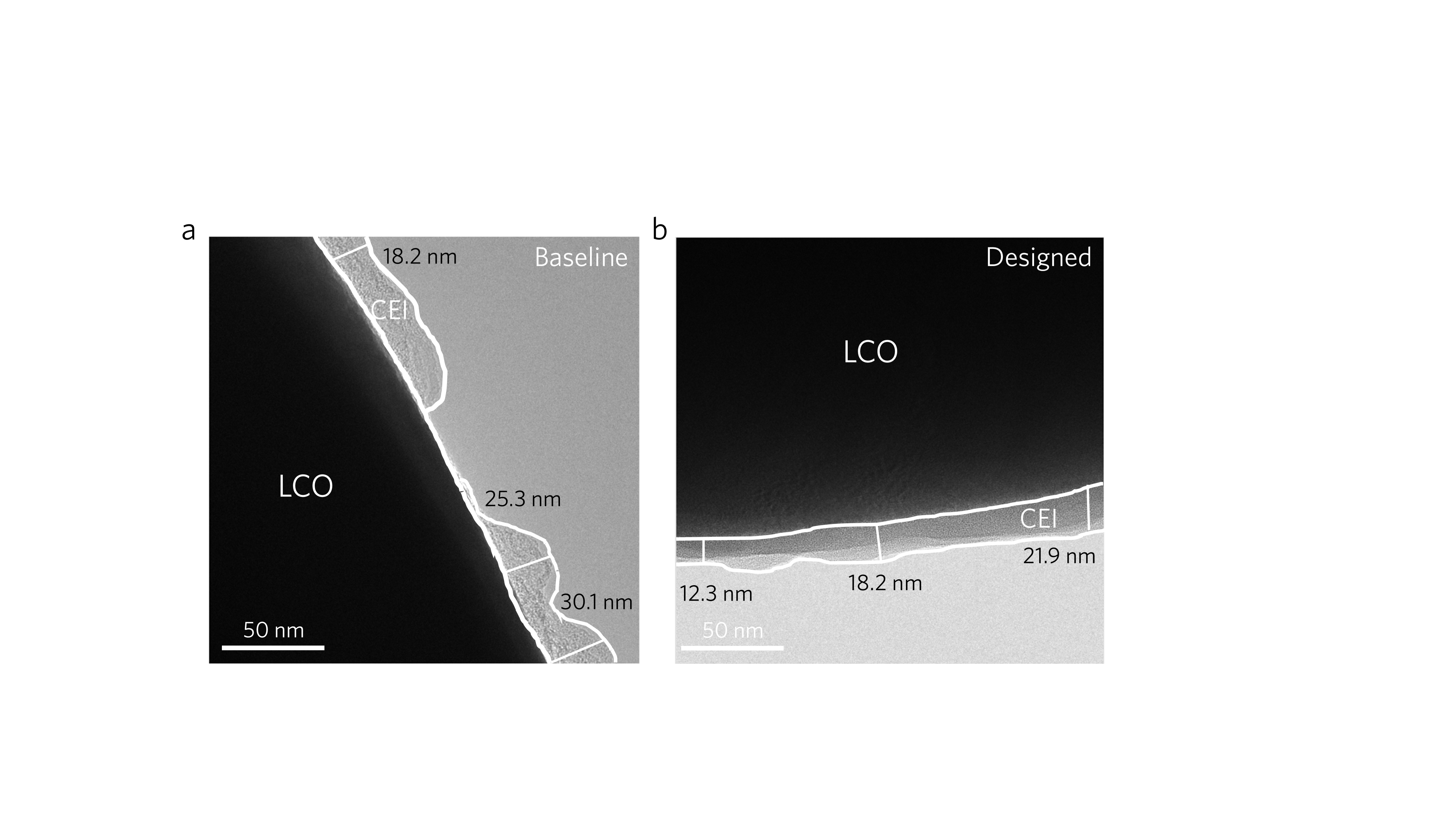


**Figure S27**. HR-TEM patterns of LCO particles cycled in (a) baseline and (b) designed electrolyte after 200 cycles.


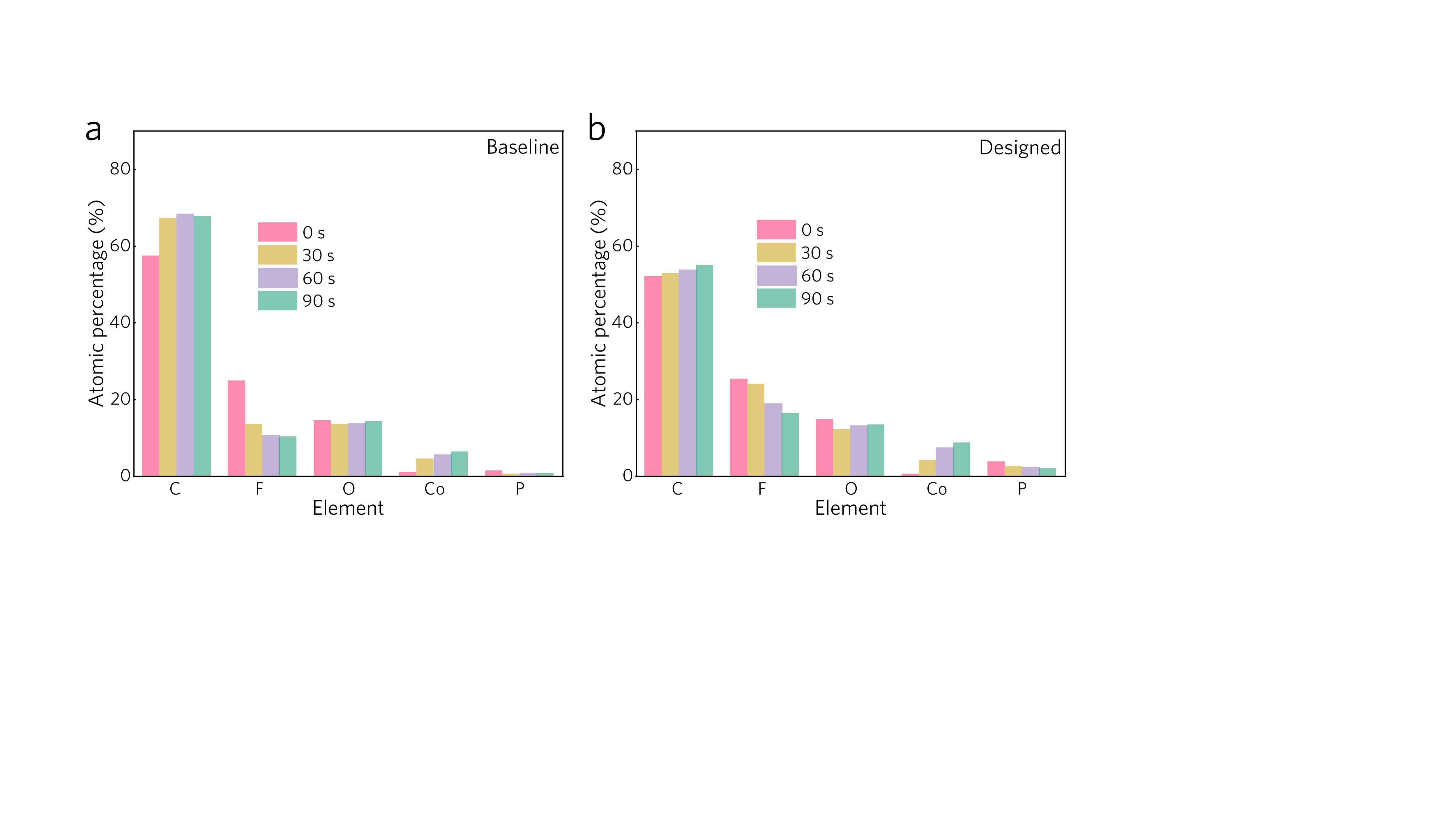


**Figure S28**. Atomic percentage of different elements on LCO surface after 200 cycles in (a) baseline and (b) designed electrolyte.

**Table S1**. Key physical properties of different carbonate solvents.

|  | Dielectric constant (25 ^o^C) | Viscosity (mPa.s) (25 ^o^C) | Freezing point (^o^C) |
| --- | --- | --- | --- |
| EC | 89.78 (40 ^o^C) | 1.90 (40 ^o^C) | 36.4 |
| PC | 64.92 | 2.53 | -48.8 |
| DMC | 3.11 (20 ^o^C) | 0.59 (20 ^o^C) | 4.6 |
| EMC | 2.96 | 0.65 | -53 |
| DEC | 2.81 | 0.75 | -74.3 |
| FEC | 79.7 (20 ^o^C) | 4.4 (20 ^o^C) | 20 |
| VC | N/A | 1.86 | 20 |

**Table S2**. Comparison between the physical and chemical properties of different solvents. ε refers to dielectric constant; η refers to viscosity; FE refers to freezing point; Li-solvent refers to Li^+^-solvent binding energy; HOMO refers to the HOMO energies of Li^+^-solvent.

|  | ε | η  (mPa.s) | FP  (^o^C) | Li-solvent  (eV) | ESP_max_ (eV) | ESP_min_ (eV) | HOMO (eV) |
| --- | --- | --- | --- | --- | --- | --- | --- |
| PC | 64.92 (25 ^o^C) | 2.53 (25 ^o^C) | -48.8 | -2.21 | 1.49 | -1.75 | -12.8 |
| EMC | 2.96 (25 ^o^C) | 0.65 (25 ^o^C) | -53 | -2.01 | 0.89 | -1.52 | -12.7 |
| DMC | 3.11 ( 20 ^o^C) | 0.59 (20 ^o^C) | 4.6 | -1.99 | 0.79 | -1.43 | -12.9 |
| FEC | 79.7 (20 ^o^C) | 4.4 (20 ^o^C) | 20 | -1.97 | 1.89 | -1.47 | -13.7 |

**Table S3**. Corresponding cell parameters and calculated energy density of assembled Gr||LCO full-cells.

| Anode material | LCO |
| --- | --- |
| Cathode material | Graphite |
| Areal loading of cathode (mg cm^-2^) | 6.6 |
| Areal loading of anode (mg cm^-2^) | 4.0 |
| Discharge mean voltage (V) | 3.94 |
| Gravimetric energy density (Wh kg⁻¹) | 303 |
| Volumetric energy density (Wh L⁻¹) | 651 |
